# Assessing arthropod diversity metrics derived from stream environmental DNA: spatiotemporal variation and paired comparisons with manual sampling

**DOI:** 10.7717/peerj.15163

**Published:** 2023-03-31

**Authors:** Aaron A. Aunins, Sara J. Mueller, Jennifer A. Fike, Robert S. Cornman

**Affiliations:** 1 Eastern Ecological Research Center, U.S. Geological Survey, Kearneysville, West Virginia, United States; 2 Wildlife and Fisheries Sciences Program, The Pennsylvania State College, State College, Pennsylvania, United States; 3 Fort Collins Science Center, U.S. Geological Survey, Fort Collins, Colorado, United States

**Keywords:** eDNA, Biomonitoring, Arthropods, Benthic invertebrates, Metabarcoding, Freshwater streams, Water quality

## Abstract

**Background:**

Benthic invertebrate (BI) surveys have been widely used to characterize freshwater environmental quality but can be challenging to implement at desired spatial scales and frequency. Environmental DNA (eDNA) allows an alternative BI survey approach, one that can potentially be implemented more rapidly and cheaply than traditional methods.

**Methods:**

We evaluated eDNA analogs of BI metrics in the Potomac River watershed of the eastern United States. We first compared arthropod diversity detected with primers targeting mitochondrial 16S (mt16S) and cytochrome c oxidase 1 (cox1 or COI) loci to that detected by manual surveys conducted in parallel. We then evaluated spatial and temporal variation in arthropod diversity metrics with repeated sampling in three focal parks. We also investigated technical factors such as filter type used to capture eDNA and PCR inhibition treatment.

**Results:**

Our results indicate that genus-level assessment of eDNA compositions is achievable at both loci with modest technical noise, although database gaps remain substantial at mt16S for regional taxa. While the specific taxa identified by eDNA did not strongly overlap with paired manual surveys, some metrics derived from eDNA compositions were rank-correlated with previously derived biological indices of environmental quality. Repeated sampling revealed statistical differences between high- and low-quality sites based on taxonomic diversity, functional diversity, and tolerance scores weighted by taxon proportions in transformed counts. We conclude that eDNA compositions are efficient and informative of stream condition. Further development and validation of scoring schemes analogous to commonly used biological indices should allow increased application of the approach to management needs.

## Introduction

Safeguarding the health and function of freshwater ecosystems is a pressing need, primarily due to widespread anthropogenic effects on flows, physical habitat, water temperature, turbidity, and chemistry, as well as introduced noxious species (Allan, 2004). Strategic allocation of resources to manage these impacts requires an accurate understanding of habitat status and trends, including whether past conservation efforts have realized anticipated gains (Karr, 1991; Pullin et al., 2013; Buss et al., 2015). To this end, assessment protocols have been developed that integrate many aspects of the freshwater environment into simple and actionable metrics of ecological integrity (Karr, 1987; Barbour et al., 1999).

A key tool for rapid assessment of ecological condition is the quantification of ‘bioindicator’ taxa, the relative abundance of which are predictably related to habitat condition (Karr, 1987; Hilsenhoff, 1987). Various biological clades have been emphasized in the development of biomonitoring strategies, ranging from fishes to diatoms (Barbour et al., 1999). While broad sampling of phylogenetic and trophic groups can increase sensitivity and illuminate ecological processes (*e.g*., Justus et al., 2010; Carlisle et al., 2008), efficiency may favor focusing on well-studied taxonomic groups with high information content and broad distributions. Benthic invertebrates (BI) as a group and arthropods as a phylogenetic clade have become preeminent indicator taxa for these reasons as well as the relative ease of their sampling and identification, allowing standardized protocols to be developed (Barbour et al., 1999; Southerland et al., 2007; Norris & Sanders, 2009). Protocols typically synthesize multiple components of BI diversity, often using threshold-based binning systems to combine disparate variables. In addition to taxonomic diversity, other variables considered by indices include ‘tolerance scores’ derived from studies of organismal susceptibility to environmental degradation (Hilsenhoff, 1987), and functional or trophic diversity. However, geographic region, physiography, and stream scale are also important covariates, which has led to a proliferation of variant protocols for specific applications (*e.g*., Roth et al., 1998; Southerland et al., 2007).

Despite success in the design of biomonitoring strategies that integrate diverse biological inputs, they remain challenging to implement at the spatial and temporal scales often needed for management. Numerous variables must be enumerated with precision, requiring large sample sizes (Mueller, 2016; Bush et al., 2019), and natural spatial and temporal heterogeneity can be large (Bush et al., 2019). These factors, coupled with user variation in collecting and identifying taxa, usually necessitate repeated sampling to differentiate sites by biological index score (Karr et al., 1986; Gibson et al., 1996) or, by extension, to detect trends at individual sites. Additionally, high-throughput taxonomic identification of benthic invertebrates remains laborious, requires specialized expertise, and is rarely revisable retrospectively (Gaston & O’Neill, 2004; Ji et al., 2013).

Environmental DNA (eDNA) metabarcoding is a recently developed, indirect approach to biomonitoring based on the detection of biomolecular residues taxa emit in their environment (Baird & Hajibabaei, 2012). It is now well-established that macroorganism DNA accumulates in various cellular and acellular states in the water column (as well as bound to sediments or incorporated into biofilms), which in principle can be isolated and genetically analyzed (Barnes & Turner, 2016). PCR-based capture of taxonomically informative ‘barcode’ genetic loci, coupled with high-throughput sequencing methods, allows a deep sampling of taxonomic clades represented in eDNA samples. The prevalence or proportion of metabarcode sequences derived from particular taxa can then be estimated, although the taxonomic resolution varies by genetic locus and the clades of interest. Previous studies applying eDNA metabarcoding to freshwater environments have associated environmentally impaired freshwater with lower BI diversity (Bagley et al., 2019; Marshall & Stepien, 2020). More recently, additional metrics have been explored such as the proportion of bioindicator taxa detected (Uchida et al., 2020), tolerance scores or related frameworks (Marshall & Stepien, 2020; Brantschen et al., 2021; Ji et al., 2022), and trophic connectedness (Robinson et al., 2022). However, there are also important drawbacks of eDNA metabarcoding, such as the co-amplification of nontarget clades that dilute the effective sequencing effort for a given objective, amplification biases that skew inferred proportions of taxa in sequence data (Elbrecht et al., 2016; Elbrecht & Leese, 2017; Krehenwinkel et al., 2017) in a manner not easily ascertained or corrected, and the competitive character of metabarcoding technologies that produce ‘closed’ compositional data that have constrained statistical interpretations and cross-study relatability (Gloor et al., 2017; Quinn et al., 2018; Lovell, Chua & McGrath, 2020). At present, the sparsity of genetic databases is an additional limitation (Hajibabaei et al., 2016) that will hopefully be transcended by continued genetic characterization of biodiversity.

eDNA biomonitoring studies have often used the mitochondrial cytochrome c oxidase 1 (cox1) gene (commonly abbreviated COI in the metabarcoding literature), and degenerate primers amplifying a subset of the original Folmer region of COI (Folmer et al., 1994) have been popular for several reasons. First, taxonomic diversity detected at COI is often higher than at other loci (Elbrecht & Leese, 2017) and quantitative relationships between source abundance and sequence proportions are sometimes seen (Krehenwinkel et al., 2017). Critically, the long-standing success of the Folmer region for phylogenetics and specimen barcoding has driven the expansion of COI reference databases (Ratnasingham & Hebert, 2007; Porter & Hajibabaei, 2018) that dwarf resources currently available for most other loci (Hajibabaei et al., 2016). On the other hand, COI primers often capture arthropod sequences at a low proportion of the total (Hajibabaei et al., 2019b)— although more targeted designs can be successful in enriching for certain clades (Leese et al., 2021)— and poor amplification success is sometimes reported (*e.g.*, Zhan et al., 2014; Mueller, 2016). In comparison, the proportion of arthropod sequences amplified by primers targeting the mitochondrial 16S ribosomal gene (mt16S) can be higher (Alberdi et al., 2018) and with comparable taxonomic breadth and resolution when reference sequences are available (Elbrecht & Leese, 2017). Furthermore, mt16S amplicons are generally shorter than COI amplicons, which is favorable for recovering degraded DNA and reducing sequencing error. Regardless of primer set, however, much uncertainty remains regarding the spatial and temporal scales of variation in arthropod compositions detectable by metabarcoding and how this might affect the interpretation or utility of BI metrics, especially since eDNA analogs of BI schemes are relatively recent (Pawlowski et al., 2018) and remain to be extensively evaluated and optimized. Empirical evaluations of metabarcoding protocols are therefore likely to remain essential for designing biomonitoring schemes in the near term. Furthermore, from the standpoint of biomonitoring precision, consistency of eDNA capture may be more important than total diversity detected, and both factors are likely to depend not only on the genetic locus (Clarke et al., 2014; Elbrecht et al., 2017) but also sampling environment (Barnes et al., 2014; Pilliod et al., 2014; Jane et al., 2015), sampling method (Majaneva et al., 2018; Sakata et al., 2021; Alberdi et al., 2018), and sequencing library protocol preparation (Zizka et al., 2019). Pawlowski et al. (2018) review many of the technical and ecological aspects of eDNA metabarcoding in relation to biological index formulation and biomonitoring.

In this study, we investigated patterns of arthropod diversity quantifiable by eDNA biomonitoring with the mt16S primers of Elbrecht et al. (2016) and the COI primers of Leray et al. (2013), which, for arthropods, amplify products with mode sizes *circa* 157 bp and 313 bp, respectively, inclusive of primers (Elbrecht et al., 2016; Elbrecht & Leese, 2017; Krehenwinkel et al., 2017). Both primer sets have high amplification rates for diverse arthropod clades (Elbrecht et al., 2016; Elbrecht & Leese, 2017; Brandon-Mong et al., 2015). Of course, the potential for detecting other biological-index taxa (*e.g*., diatoms, fish, and bivalves) may factor into marker choice, but in the present study we focus exclusively on comparing arthropod diversity.

Our study surveyed units of the U.S. National Park System (NPS) in the Potomac River watershed of the mid-Atlantic seaboard, for which past biomonitoring records (*e.g*., Klauda et al., 1998; Lookingbill et al., 2014; Mueller, 2016) allow methodological comparison. The study was divided into two distinct stages. The first stage consisted of paired sampling events at 13 diverse sites within nine parks, in which eDNA was collected in parallel with the manual BI inventories described in Mueller (2016). This stage had three main objectives: (1) to bolster records of taxa present in the watershed, achieved by increasing sampling depth relative to previous work (Mueller, 2016); (2) to sequence a subset of manually identified specimens at the mt16S locus to help validate our taxonomic assignment method, given that this locus is more sparsely represented in public databases compared to the COI locus (Hajibabaei et al., 2016); and (3) to directly compare, through coordinated collection, taxonomic overlap between manual and eDNA surveys performed in concert as well as the similarity of metrics derived from those methodologies.

In the second stage of our study, our main objective was to evaluate spatial and temporal variation in metrics of arthropod diversity by intensively sampling three NPS units. Five rounds of eDNA sampling were performed at 5–6 sites within each park. In this second stage we specifically sought to assess whether metrics derived from repeated eDNA sampling actually distinguished sites previously shown to differ by BI scores. The study sites differ with respect to stream condition as well as urban land cover (Lookingbill et al., 2014), as the latter has been shown to be a major determinant of arthropod diversity and biological index scores (Vølstad et al., 2003; Goetz & Fiske, 2008; Utz, Hilderbrand & Boward, 2009; Stranko, Hilderbrand & Palmer, 2012; Lookingbill et al., 2014) as well as water quality (Tong & Chen, 2002). We compared stream reaches using metrics such as genus-level alpha diversity and the proportions of key indicator groups such as chironomids and “EPT” taxa, *i.e.*, insects within orders Ephemeroptera (mayflies), Plecoptera (stoneflies), and Trichoptera (caddisflies). EPT taxa are strongly associated with stream benthos during much of their development, perform key ecological functions, and have longstanding service as ecological indicators (Hilsenhoff, 1987; Emilson et al., 2017). We also adapted existing classifications of tolerance and functional group appropriate to the region (Norris & Sanders, 2009) to generate tolerance scores for each eDNA sample weighted by the inferred proportions of taxa. Secondarily, we sought to identify factors that affect eDNA recovery in these environments, such as barcode locus, season, PCR inhibition, and filter type, for further protocol optimization.

## Materials and Methods

### Paired manual and eDNA surveys

**Figure 1:**
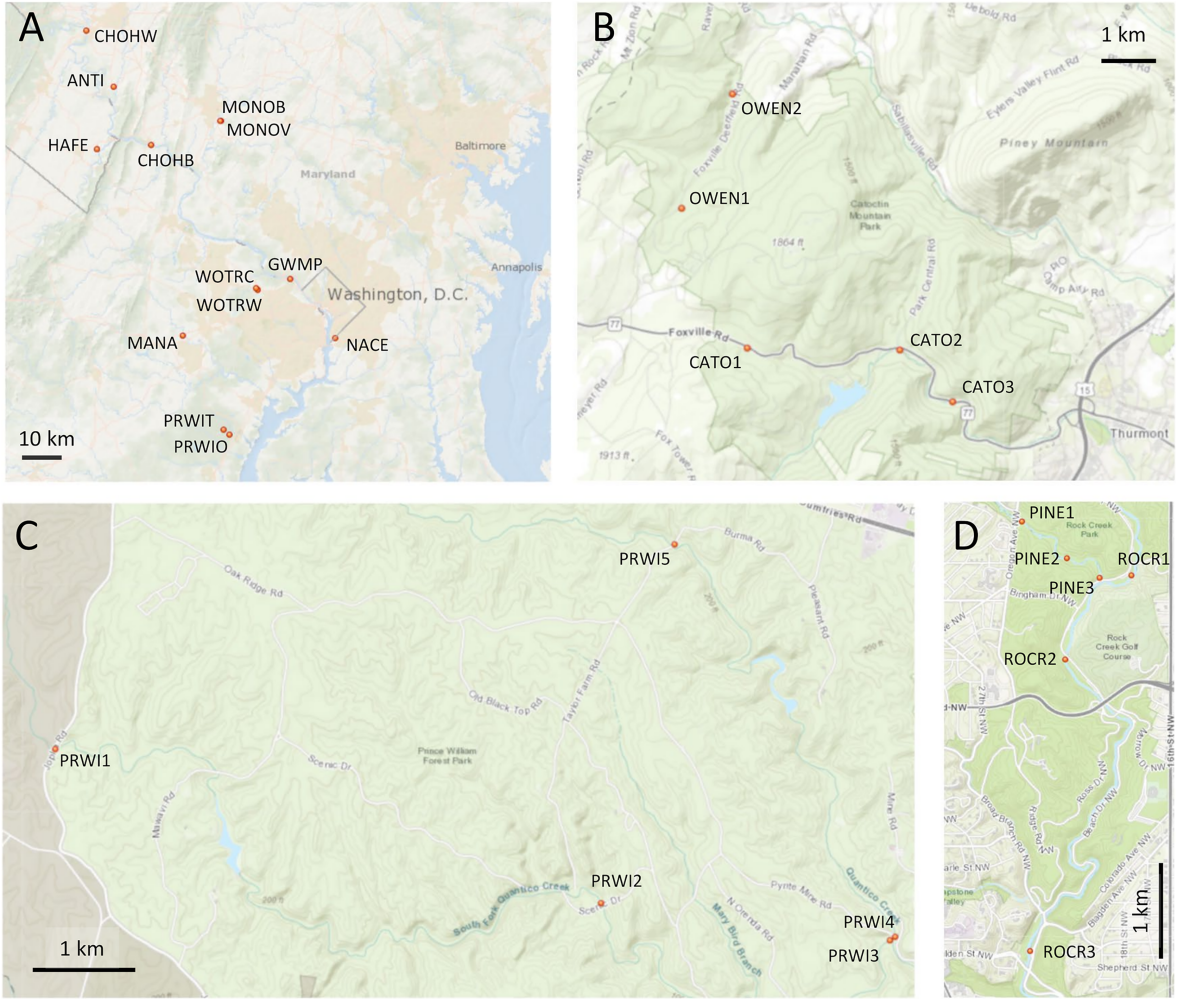
Maps of sampling locations. Site identifiers used in text are shown. (A) Thirteen sites at which paired environmental DNA (eDNA) and manual sampling was performed. (B) Sites of repeated eDNA sampling within Catoctin Mountain Park, Maryland. (C) Sites of repeated sampling within Prince William Forest Park, Virginia. (D) Sites of repeated sampling within Rock Creek Park, District of Columbia. Map generated with the National Map Viewer tool (https://apps.nationalmap.gov/viewer/).

Paired sampling occurred in 2015 at units of the National Park Service (Fig. 1A), with permission, as detailed in Mueller (2016). Site metadata are provided in File S1 and more detailed information is available in Norris & Sanders (2009). Counts of taxa identified manually were previously reported in Mueller (2016) and generated following protocols developed by the NPS for the region as described therein. In this first stage, we tested eDNA sampling using both plankton net and grab bottles. However, as the plankton-net samples typically yielded lower diversity than grab samples they are not further analyzed here.

eDNA-bearing particles were filtered from 500 mL of each grab sample using 47 mm-diameter cellulose nitrate filters with a 0.45 µm pore size (Sartorius Stedim PN 1130647ACN). Filtering used a 2 liter vacuum flask with either a Pall magnetic filter funnel or a glass Sibata filter funnel. Filter funnels were cleaned between each sample by soaking them in a solution of 10% bleach for 15 min, followed by 1 min of rinsing with MilliQ-treated DNAse-free water (Millipore) to remove residual bleach. DNA was then extracted from filters using the Qiagen DNEasy PowerWater kit per manufacturer’s instructions with the exception of decreasing the final elution volume to 75 µl. All eDNA extractions for both stages of the study were performed in laboratory space dedicated to the extraction of samples with low template quantity, including rigorous cleaning procedures and a strict exclusion of post-PCR materials to limit cross-contamination.

### Sequencing library preparation (first stage)

Amplicon sequencing libraries were generated for the Illumina MiSeq following the manufacturer’s protocol for Nextera multiplexing (Illumina, 2016) with minor modifications, in which the initial amplification was performed with the base primers extended with the Illumina-specified adapter sequences. Each locus was amplified in triplicate 11.5 µl reactions consisting of 1 µl template, 0.5 µl of each primer (5 µM each), 6.25 µl KAPA HiFi Master Mix, and 3.75 µl PCR grade water. Thermocycling parameters were 95 °C for 3 min, 35 cycles of 95 °C for 30 s, 59 °C (COI) or 62 °C (mt16S) for 30 s, 72 °C for 30 s, and a final extension at 72 °C for 5 min. The triplicate reactions were pooled for each amplicon, and 25 µl of the combined product was used for AMPure bead cleanup as described in the Illumina protocol. Samples were dual indexed with Illumina Nextera indexes, cleaned with AMPure beads following Illumina (2016), and manually diluted to a concentration of 4 nM. Five µl aliquots of each diluted library were pooled. The final library consisting of both the field collected eDNA samples and mocks (see below) were loaded onto a 600 cycle v3 kit at 12 pM with 10% phi-X and sequenced for 300 cycles in each direction.

### Field sampling (second stage)

Three parks were selected in consultation with the NPS for repeated sampling (Figs. 1B–1D): Prince William Forest Park, Catoctin Mountain Park, and Rock Creek Park. Within Catoctin Mountain Park, we sampled Big Hunting Creek and Owens Creek, which are distinct drainages that do not merge within the park, and are designated CATO and OWEN, respectively. In Rock Creek Park, we sampled Pinehurst Branch and Rock Creek, the former at the specific request of the NPS due to anticipated restoration work therein (https://doee.dc.gov/service/pinehurst-stream-restoration-project). Pinehurst Branch is a small stream assigned a 14-digit hydrologic unit code (HUC), which is 1.3 miles in length, has a 619-acre catchment (https://doee.dc.gov/service/pinehurst-stream-restoration-project), and experiences intermittent flow during drier periods. It is a tributary of the much larger Rock Creek, which has an 8-digit HUC and a catchment of 76.5 square miles. These reaches are designated PINE and ROCR, respectively, and, while forested within Rock Creek Park, they are embedded within a highly urban landscape (Carruthers et al., 2009). Within Prince William Forest Park, we sampled the south and main forks of Quantico Creek, which, since they are very similar in scale and physical habitat, were both given the same reach designation (PRWI). Prince William Forest Park and Catoctin Mountain Park are forested and embedded in a largely rural landscape, and stream quality in those parks scored high in the assessment of Lookingbill et al. (2014), whereas stream condition was degraded in Rock Creek Park. NPS resource assessments also list ROCR and PINE as degraded relative to the other sampled areas. Benthic invertebrate biological index (BIBI) scores previously reported for Prince William Forest Park and Catoctin Mountain Park averaged 3.96 and 4.00, respectively, out of a maximum of 5 (Nortrup, 2015; Walsh et al., 2015). The average score reported for ROCR (Nortrup, 2013) was 1.67 (the theoretical minimum is 1).

Based on our own observations and other data, we considered two sites to be potentially degraded relative to other sites within the same reach, namely PRWI1 and CATO2. PRWI1 was situated on the park boundary at a high-traffic bridge crossing that was visibly more sedimented than other PRWI sites, which can have a significant effect on benthic invertebrates (Zweig & Rabeni, 2001). CATO2 was sited approximately 1 km downstream of a small lake, was in the vicinity of parking lot construction that we observed at the time of sampling and had previously been given a poor fish biological index (FIBI) score by the NPS, although the BIBI score generated by the NPS was similar to other sites in the park (Thomas et al., 2013). A previous study had found a high incidence of dermatocystidiosis in native sculpin and reduced numbers of that sensitive species in the immediate vicinity of CATO2 but not in Owens Creek (Blazer et al., 2016). We therefore make specific note in the Results of the extent to which these two sites were differentiated by arthropod metrics relative to other sites within the same reach.

Three rounds of collections were made approximately monthly from July to September of 2019, and an additional two rounds were performed approximately 6 weeks apart in spring 2020. The exact timing of sampling was not strictly defined in advance to allow for contingencies such as staff availability, COVID-19 restrictions, and unfavorable field conditions. No sampling was performed within a day of significant rainfall. Collections were performed under permits CATO-2019-SCI-0009, PRWI-2019-SCI-0010, and ROCR-2019-SCI-0019. Collection metadata are tabulated in File S2.

Prior to traveling to field sites, reusable equipment was decontaminated and negative controls were prepared for transport to the field. Polypropylene filter holders (47 mm diameter, Advantec) with nylon male barb fittings attached to each end (Cole Parmer) were disassembled and soaked in a 10% bleach solution for 10 min. Using gloved hands and without touching the filter-contacting faces, the filter holder components were then rinsed under a DNA-free MilliQ water dispenser for 1 min to remove residual bleach. The rinsed components were placed in a laminar flow hood and the filter-contacting surfaces exposed to UV for 30 min. Decontamination occurred in a laboratory space from which PCR products were excluded. The filter holders were then loosely assembled and placed in Whirl-Pak bags for transport to field sites. Negative-control samples consisted of 1 L DNA-free MilliQ water. Prior to use, sample containers were decontaminated in the laboratory by soaking 1 L polypropylene bottles with a solution of 10% bleach for 30 min. The lids were placed loosely on the bottles, which were gently shaken twice during the 30 min soak period. The bottles were rinsed by discarding the bleach solution and then rinsing six times by filling them half full with DNA-free MilliQ water, capping loosely, shaking, and discarding.

Sampling was always performed with gloved hands. At each site, a 10-ft section of Tygon pump tubing (Masterflex) was cut from the stock roll (tubing cutters had been sanitized in the laboratory using the process described above for the filter holders). The tubing was threaded through a Geotech peristaltic pump and attached to the intake of a sanitized filter holder. The peristaltic pump was configured to draw water *via* suction through the tubing intake, across the filter, and out of the filter-holder outlet. If the site was the first-visited within a stream reach, a negative-control sample was filtered by placing the tubing intake untouched into the bottle and filtering the entire contents. All filters were handled with single-use forceps, and after use each was placed into a sterile 15 mL plastic screw-cap tube and then into a Whirl-Pak bag for secondary containment. Filters were stored in a cooler with icepacks until transport back to the lab, at which point they were placed into a −80 °C freezer until extraction. All samples were returned from the field within 8 h.

After control sample collection, the tubing was anchored to a branch with a zip tie and placed in flowing stream water, thereby securing the tubing intake and directing it upstream and away from the branch. The tubing and empty filter holder were first flushed with stream water for 2 min before the first environmental filter was added. The filtrate volume was measured by directing the outlet of the filter into a graduated cylinder. For replicate samples at a single site, no additional tubing flushes with stream water were performed. Because stream reaches are already connected biologically, the same tubing and filter holder was reused within the same stream reach (*e.g*., CATO1, CATO2, and CATO3) after flushing with stream water for 2 min at the new site. For each new stream reach, a new section of tubing was used with a new decontaminated filter holder.

A targeted maximum of one liter of water was filtered through each filter, but this target was not always achieved due to gradual blockage of the filters (see Results), an anticipated result that implies locally higher concentrations of suspended particles. Two biological-replicate filters were obtained back-to-back to ensure adequate DNA recovery, but these were subsequently pooled for most analyses (see below). All sites were sampled using 0.45 cellulose nitrate (CN) filters (MDI), which have been reported to perform well for eDNA capture (Goldberg et al., 2011). However, as eDNA compositions can be affected by filter type (Barnes & Turner, 2016; Goldberg et al., 2016), for comparison we also obtained replicate samples using 0.2 µm CN (MDI), and 0.8 µm mixed cellulose ester (MCE) filters (Advantec) at a subset of sites during the third round of sampling (in September 2019). Filter comparisons were not performed at PRWI due to a higher level of suspended particulate that prevented use of the 0.2 µm CN filters.

### Sequencing library preparation (second stage)

DNA was extracted with the Qiagen DNEasy Kit, largely following the procedure of Renshaw et al. (2015) but with slight modifications described below. Whole filters were transferred into 5 mL screw-cap tubes using single-use sterile plastic forceps. Then 567 µl of ATL buffer (Qiagen) and 63 µl of Proteinase K (Qiagen) were added to each tube. Lysis tubes were vortexed at low speed for 5 min at room temperature to mix the contents and then incubated at 56 °C for a total of 30 min. Halfway through the incubation period, samples were removed and vortexed at high speed for 5 min at room temperature before being returned to the incubation chamber. After the 30-min incubation, samples were vortexed again at low speed for 5 min and then 630 µl of AL buffer (Qiagen) was added to each tube. The tubes were then incubated at 56 °C for an additional 10 min, after which 630 µl of 95% ethanol was added to each. The entire volume of lysate was applied to a single column in multiple aliquots, each of which were passed through *via* centrifugation at the recommended force, and the pass-through volumes discarded. Once the entire lysate volume was applied to the spin column, the remainder of the extraction protocol followed the manufacturer’s guidelines, with the exception that an elution volume of 125 µl was used. DNA samples were stored frozen at −20 °C until shipment to the Fort Collins Science Center, Fort Collins, Colorado for further processing.

At all steps of library preparation, DNA concentration was quantified with the Qubit dsDNA High-Sensitivity Assay following the manufacturer’s protocol. After each library PCR, reaction aliquots were electrophoresed in 2% agarose gels and visualized with ethidium bromide to confirm the target amplicon. All reaction products were cleaned of residual reagents using the Qiagen UltraClean-htp 96 Well PCR Clean-up Kit and eluted in 30 µL water.

Extracts from the two replicate filters obtained at each single sampling event were pooled prior to the inhibitor clean-up step, except for technical replicate pairs. Initially, all DNA extracts were cleaned using the Zymo OneStep PCR Inhibitor Removal Kit, whereas a subset of samples was also cleaned with the Qiagen DNEasy PowerClean kit for comparison. Both kits were implemented per manufacturer’s instructions. As the sequencing for this stage was performed on two MiSeq flowcells, we compared the Zymo and Qiagen kit replicates on the first chip and, given that the Qiagen kits performed better (see Results), used the remainder of the Qiagen kit (50 preps total) for stream reaches showing the greatest benefit from that kit in the test (see Results).

In contrast to the first stage of paired sampling, library preparation for the repeated sampling stage used an initial ‘preamplification’ to increase the concentration of the target loci relative to other DNA fragments (Hajibabaei et al., 2019b). Preamplification PCRs were performed in triplicate with base primers lacking Illumina extensions and the reaction products pooled to reduce stochastic variation (Polz & Cavanaugh, 1998). Preamplifications were performed with Promega GoTaq Flexi Taq and PCR master mix following the manufacturer’s instructions. The thermocycler program included a preheat of 95 °C for 2 min, then 25 cycles of 95 °C for 45 s, an annealing temperature of 50 °C for 45 s, and 72 °C for 45 s. A final extension cycle of 72 °C was performed for 2 min.

Product from the preamplifcation reaction was then input into the recommended library protocol already described, *i.e*., the addition of Illumina-specific adapters and the addition of dual indexes in sequential PCRs. The first library amplification used the KAPA 2G polymerase and master mix following manufacturer’s instructions. The thermocycler program was the same as for preamplification, except that a common annealing temperature of 50 °C was used, since the incorporation of long adaptor sequences reduces the thermodynamic differences between locus-specific primers. The indexing PCR used the KAPA HiFi HotStart high-fidelity enzyme and reagent mix following manufacturer’s instructions. The thermocycler program specified an initial 3-min preheat at 95 °C, 8 cycles of 95 °C, 55 °C, and 72 °C (30 s for each step), and a final extension at 72 °C for 5 min.

Libraries were quantified and pooled equimolarly, after which a 30% PhiX spike was added. The pooled libraries were diluted to a final concentration of 4 pM before loading on a MiSeq v.3 600-cycle chip and sequencing in each direction for 250 cycles. Laboratory and field negative controls were extracted and quantified independently but sequenced as two pools, one for each type.

### Reference database generation

All arthropod sequences available in the nucleotide (nt) database of the National Center for Biotechnology Information (NCBI) were downloaded on January 11, 2022 and filtered to include only mitochondrial sequences less than 50 kb. All genera listed in the Maryland Biological Stream Survey (MBSS) data (Maryland Department of Natural Resources, 2021), as well as all genera listed in Mueller (2016) were searched for corresponding taxonomic identifiers in the NCBI Taxonomy database (available at https://ftp.ncbi.nlm.nih.gov/pub/taxonomy). These text-based matches were then confirmed to be arthropod genera by progressing through the parent taxon identifiers; this step is required because genus names are not unique and may also occur in non-arthropod clades, potentially leading to the extraction of incorrect taxonomic IDs. Genus names lacking matches in the taxonomy database were checked for spelling errors in the sources or possible synonyms. Since chironomids were not typically identified to genus in these sources, we included all children of family Chironomidae (TaxID 7149). The final list of taxonomic identifiers used is provided in File S3, for which all species-level children were extracted from the NCBI Taxonomy database.

Taxonomy-filtered arthropod sequences were then used as queries to identify their best-match coordinates to mt16S and COI sequences in our data, if any. To reduce the search time for this step, all primer-matched mt16S and COI reads (see below) were clustered at 99% with swarm (Mahé et al., 2015), for this analysis only. We did not expect all arthropod sequences to actually be represented in our data, but rather that there would be environmental matches sufficiently close to define and extract the target locus from the original NCBI accessions. This method was chosen over an *in silico* PCR approach to allow partial matches to be captured and because templates that appear to be poor matches to a primer set may nonetheless appear in amplicon data sets (Zafeiropoulos et al., 2021). However, we later filtered both our sequence data and candidate reference sequences by requiring both primers of a locus to be present, albeit partially and with error tolerance, as described below.

Alignments of reference sequences to swarm clusters were performed with BLASTN using default search parameters but with low-complexity masking disabled. Each NCBI sequence was parsed on the coordinates of its best match, after which parsed sequences were aggregated by taxon identifier and dereplicated with vsearch (Rognes et al., 2016) at 100% and the “iddef” switch set to 2. Reference sequences were then formatted for use with SINTAX (Edgar, 2016) as described in the vsearch documentation. Leave-one-out cross-validation (LOOCV) was performed by sequentially removing one sequence from the database and classifying it with the remaining sequences using SINTAX as implemented in vsearch. Additional validation was performed by assigning the mt16S voucher sequences reported in Mueller (2016), as described in the Results. The formatted databases are provided in Files S4 and S5. Primer amplification bias in mock samples and validation of taxonomic-assignment methods are summarized in Files S6–S8.

### Processing of high-throughput sequence data

Because mt16S and COI libraries for repeated sampling were not separately multiplexed for each sample, the two loci were separated by searching for expected primer sequences within the first 30 bases of each forward read with BBduk (sourceforge.net/projects/bbmap/). The kmer length for these searches was set to 19 for the COI primer and 17 for the shorter mt16S primer, whereas the kmer length at read edges (“mink” parameter) was set to 15 and 13, respectively. Iterative searches showed that lower kmer lengths frequently rejected many valid matches, whereas no increase in the number of primer-matched sequences occurred with higher kmer values. For COI, read pairs were merged using vsearch with a minimum overlap threshold of 80 and a maximum divergence threshold of 7.5%. For mt16S, the second read of each pair was discarded, as the entire locus is present in the forward read, which generally has fewer sequencing errors than the reverse (Schirmer et al., 2015). Demultiplexed reads can be retrieved from NCBI through BioProject accession PRJNA877240.

Reads were clustered into amplicon variants using swarm with default parameters, from which a table was generated with the size of each unique cluster of reads recovered in each sample. The filtered cluster representatives were taxonomically assigned with the SINTAX databases and the lowest-rank assignment with a bootstrap score of 0.9 or higher was selected. The sizes of clusters with the same taxonomic classification were then summed together to produce a taxon-level counts table. We imposed two additional quality-control measures for a cluster to be assigned within phylum Arthropoda. First, cluster representatives were required to be within specific length thresholds: more than 140 bp but less than 180 bp for mt16S, and at least 250 bp for COI. Second, cluster representatives were required to be consistent with a placement in phylum Arthropoda by a secondary method. For mt16S sequences, we searched against the nt database with BLASTN as described above and determined whether the lowest common ancestor of all matches within 3% of the top match score fell within Arthropoda. For COI cluster representatives, we used the RDP classifier (Wang et al., 2007) with the arthropod-rich COIv.4 training set of Porter & Hajibabaei (2018).

### Data censoring and transformation

We checked for contamination by plotting the sequence counts attributed to each taxon in negative control samples *vs*. in biological samples, to determine whether any unexpected taxa were present in control samples above the level consistent with sequencing “crosstalk”, which occurs when reads are assigned to the wrong sample by the demultiplexing software (MacConaill et al., 2018). No arthropod taxa were detected in pooled negative controls above ~0.01%, consistent with a lack of contamination above crosstalk levels in either the field or laboratory (Fig. S1A). In the mock samples, taxa inconsistent with the six input specimens were found at a rate consistent with a 0.01% crosstalk rate, with two potential exceptions highlighted (Fig. S1B): order Odonata at COI and genus *Climacia* at mt16S. We chose not to remove these taxa as contaminants because in the former case, no comparisons were made using reads classified above family, and in the latter case, only a single biological sample contained *Climacia* and at a relative rate tenfold higher than in the mock, which is inconsistent with an origin by contamination in the biological sample. Based on these results and MacConaill et al. (2018), we therefore censored the raw counts tables at a rate of 1 in 10,000 reads. First, the sum of raw read counts assigned to each taxon across all samples was multiplied by 0.01% and this value subtracted from all samples for that taxon. Similarly, the sum of raw read counts for all taxa within a sample was multiplied by 0.01% and this value subtracted from all taxa within that sample. Cell values were then rounded down to integer values and negative values set to zero.

To allow linear statistical analysis of compositional data, such as correlation and principal component analysis (PCA) of pairwise dissimilarity measures, the censored counts were transformed using a log-ratio approach (see Gloor et al., 2017; Quinn et al., 2018; Lovell, Chua & McGrath, 2020). For this transformation, censored values were converted to proportions within samples, scaled to the geometric mean of all proportions within that sample, and the log10 of that ratio taken for nonzero values. Because values for taxa below the mean proportion in a sample are negative after this procedure, the resulting matrix was made non-negative by adding a common scalar to each nonzero cell of the table such that the final nonzero minimum value was 0.01 (control samples were subsequently removed). This transformation was performed separately for each locus and separately for each of the two study stages. The raw counts and log-ratio transformed compositions are given in Files S9 and S10. A worked example of the log-ratio transformation we performed is given in File S11.

### Generation of genus-level tolerance scores and functional classifications

The scores and classifications of regional taxa found in Norris & Sanders (2009) were modified as follows. Taxon-specific tolerance scores were inverted from a 0–10 scale with 0 indicating highest sensitivity to a 1–11 score with 11 indicating highest sensitivity. These values were then averaged over all species within a genus, if not already scored at the genus level, and taxonomic ranks above genus were ignored. The sample-level tolerance score was then calculated as the sum of all genus-level tolerance scores multiplied by their proportion in the sample (ignoring genera with no score). Functional classifications were also extracted for all listed arthropod taxa and a single classification assigned to each genus if that classification was given only at the genus level or to a majority of species within the genus. We then limited our analysis to the five most common functional groups (scraper, gatherer/collector, predator, filterer, and shredder) as other groups listed in Norris & Sanders (2009) were very rare or absent in our data.

To compare metrics derived from paired sampling with previous stream quality assessments, we extracted and averaged NPS-reported BIBI values for the closest sampling location to each of our sites from the relevant park assessment reports listed in File S1. By design, most of our sites were approximately coincident with those used in the cited assessment reports, except for our sites within the Chesapeake and Ohio Canal NHP (CHOHB and CHOHW).

### Statistical analysis

The software package PAST (Hammer, Harper & Ryan, 2001) was used for most statistical analyses, including correlation, PCA, diversity calculations, and Kruskal-Wallis tests of medians. Detailed input and output data for Kruskal-Wallis tests and Spearman rank correlations are reported in File S12. Taxonomic diversity of manual samples was taken from Mueller (2016) and used Brillouin’s index, whereas all other diversity metrics were based on proportions and used Shannon’s index. The R package vegan (Oksanen et al., 2019) was used for rarefaction analysis. Neighbor-joining dendrograms of select mt16S sequences (see Results) were generated in MegaX (Kumar et al., 2018) using a gamma distribution of rate variation specified with five categories and a rate parameter of 1.0.

## Results

### Taxonomic comparison of paired manual and eDNA samples

Arthropod taxa detected manually and with eDNA were compared at the genus level, with the exception of chironomids, which were compared at the family level since the manual surveys did not differentiate chironomids below family level. Similar numbers of taxa were detected overall by manual surveys and COI metabarcoding, whereas fewer taxa were detected with mt16S (Fig. S2). Rarefaction curves (Fig. S3) indicate that eDNA richness at each locus was near saturation whereas curves for manual surveys indicate that new taxa were still accumulating at this level of effort. Taxa that had higher prevalence in manual surveys were also more likely to be detected at COI than mt16S (Fig. S2). However, only 6 of 86 COI taxa (7.0%) were found in a majority of the thirteen samples, compared with 11 of 43 mt16S taxa (25.6%). In the manual surveys, nine of 87 taxa were found in a majority of samples (10.3%). At the site level, overlap among taxa detected by eDNA *vs*. manual sampling was generally low but again greater at COI than mt16S (Fig. S4).

**Figure 2:**
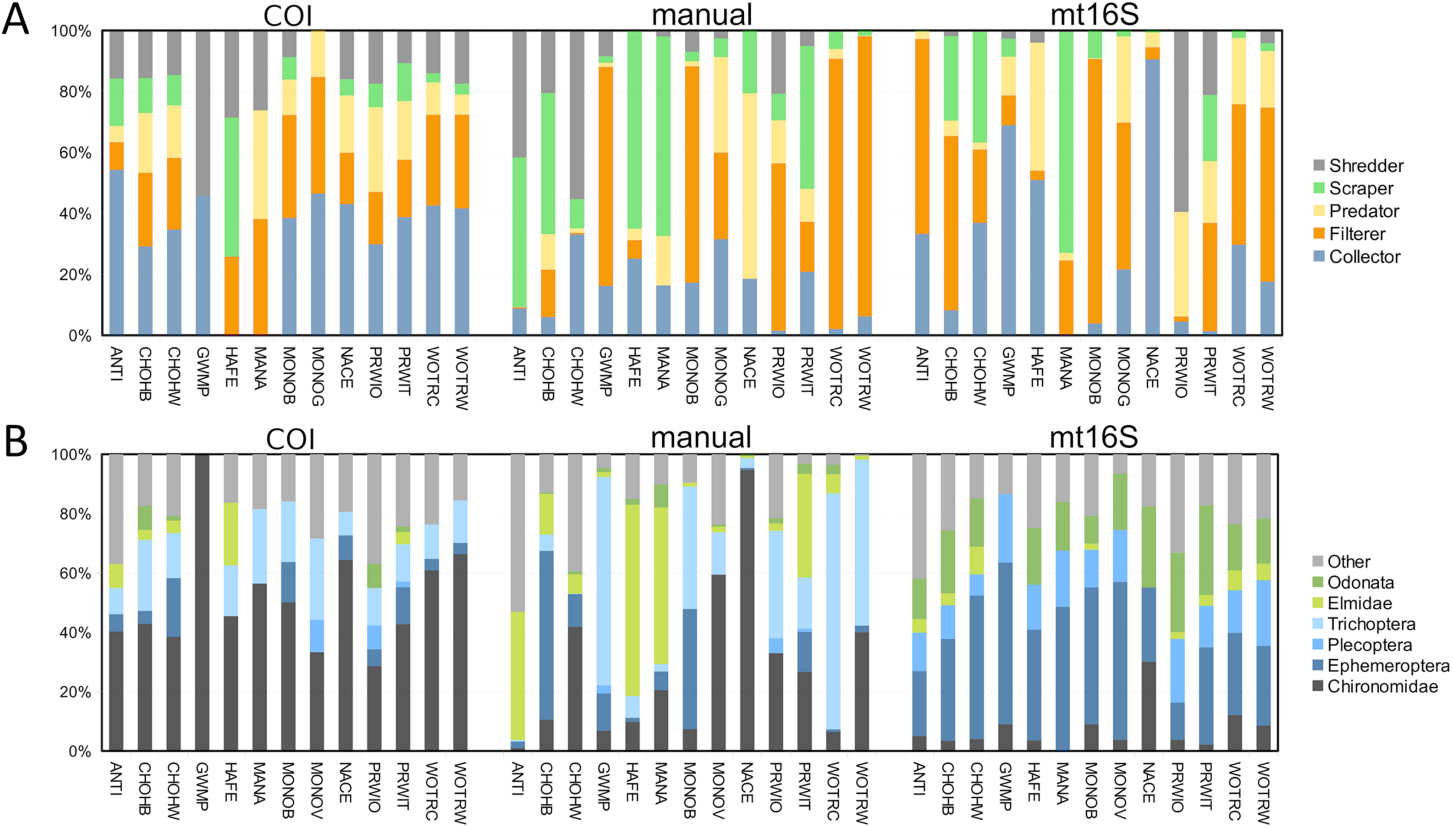
Variation in the proportions of arthropod functional and taxonomic groups represented in paired environmental DNA (eDNA) and manual surveys at thirteen sites. COI and mt16S eDNA proportions are derived from log-ratio transformed sequence counts, whereas manual proportions are derived from counts of individuals. (A) Proportions of five functional groups commonly used in benthic invertebrate indices, grouped by method. (B) Proportions of six arthropod taxa commonly used in benthic invertebrate indices, grouped by method. COI, cytochrome c oxidase 1; mt16S, mitochondrial 16S.

In addition to differing in total diversity, the two barcode loci captured distinct compositions of functional groups and key indicator taxa such as EPT (Fig. 2). The COI locus was less variable across sites with respect to functional-group proportions and had higher amounts of shredders and collectors. The COI locus captured a higher proportion of chironomids than mt16S, whereas the latter detected a high proportion of Ephemeroptera. Repeated sampling confirmed these consistent differences in composition (Fig. S5), but additionally illustrated a qualitatively bimodal pattern in mt16S compositions between the degraded reaches PINE and ROCR and other reaches, whereas among-reach differences were more subtle at COI.

Of the three arthropod metrics (taxon diversity, functional diversity, and tolerance) generated for manual, mt16S, and COI compositions, functional diversity at COI was most strongly correlated with previously derived BIBI scores at or near the same sampling locations (Fig. S6). Spearman’s rank correlation between these two variables was significant (r = 0.764, unadjusted *P* = 0.00623, *n* = 11 sites; see File S1 and Materials and Methods for details). Since many of these pairwise associations are expected to be intrinsically correlated, we did not perform any adjustment for multiple testing and thus the significant tests shown in Fig. S6 should be interpreted with caution (see File S12 for exact test results). All three manually derived metrics were positively correlated with one or both tolerance metrics derived by eDNA. Functional diversity at mt16S showed no positive association with any other metric.

### DNA yield

The second stage of our study included an evaluation of factors potentially affecting the overall cost and utility of a biomonitoring protocol. DNA yield per 0.45 µm CN filter ranged from 0.09–17.5 ng/µL with a mean of 4.78 ng/µL and appeared to be independent of volume filtered (Fig. S7), which is expected given that eDNA yield derives from the amount of material impregnated in the filter and not the filtrate volume *per se*. That is, failing to achieve the target water volume is a consequence of more rapid saturation of the filter in streams with higher particulate level and should not be interpreted as a lower sampling effort. The 0.45 µm CN filters performed well compared to the 0.8 µm MCE and 0.2 µm CN filters (Fig. S8). The DNA yield for 0.45 µm CN filters was more consistent than for the other filters, and higher than both within reaches yielding generally lower levels of total DNA per sample. Indeed, the 0.2 µm filters were effectively nonfunctional at PRWI due to the visibly higher particulate levels in that reach. The greater consistency of 0.45 µm CN filters and higher yield at more challenging sites is advantageous as it reduces uncertainty and thus the time and resources needed in the field to ensure sampling targets are met.

### Relative rate of arthropod recovery

The sample-averaged proportion of all reads assigned to an arthropod genus or to family Chironomidae was higher for mt16S (14.0%) than for COI (2.53%) (non-arthropod reads constituted the large majority for both loci, as expected). Overall variation among samples was similar for both primer sets (CVs of 0.78 and 0.75, respectively), but both primer sets varied in arthropod recovery among reaches (Fig. S9), with lower recovery rates at the degraded ROCR and PINE sites relative to PRWI. However, eDNA capture method appears to influence these variables as well: for example, while the recovery rate at COI with the method used in paired sampling (averaging 2.72%) was very similar to the recovery rate in repeated sampling, it was substantially lower at mt16S (averaging only 3.81%). In the second stage (repeated sampling), the proportion of COI reads assigned to phylum Arthropoda at any rank (2.7%) was similar to that reported by Hajibabaei et al. (2019b) with the same COI barcode (2.9%), although those authors used the inosine-based modification suggested by Geller et al. (2013).

### Technical replication

Replicates were compared after removing very rare taxa (present in less than three of 117 total samples in the repeated sampling stage). Pearson correlation coefficients of transformed taxon proportions between replicates were generally greater than 0.8 (Fig. S10), although lower coefficients were seen for a few COI replicates when total read counts were low or diversity was high. Pearson correlation coefficients were higher overall for the mt16S barcode than the COI barcode. Strict technical replicates of the same biological sample had correlation coefficients comparable to those of technical replicates using different inhibition-removal kits, biological replicates using the same filter type, and biological replicates using different filters. These observations imply that the tested technical factors had minimal impact on taxonomic compositions. Technical replication is therefore more likely affected by stochastic factors that cause dropout of taxa recovered at lower levels in the data (see Fig. S11). Since we did not see any impact of filter type or inhibition kit on taxonomic compositions, we included all replicate types in our diversity analyses.

The total number of reads obtained from Zymo-processed replicates was in all cases fewer than for Qiagen-processed samples, with an average ratio of 0.541 (0.265 SD, *n* = 11). Stochastic variation in library preparation and loading could have contributed to some of this difference, but the consistency of the observation indicates that the differences were primarily attributable to the inhibition-removal kit used.

### Arthropod diversity metrics within and among reaches with repeated sampling

**Figure 3:**
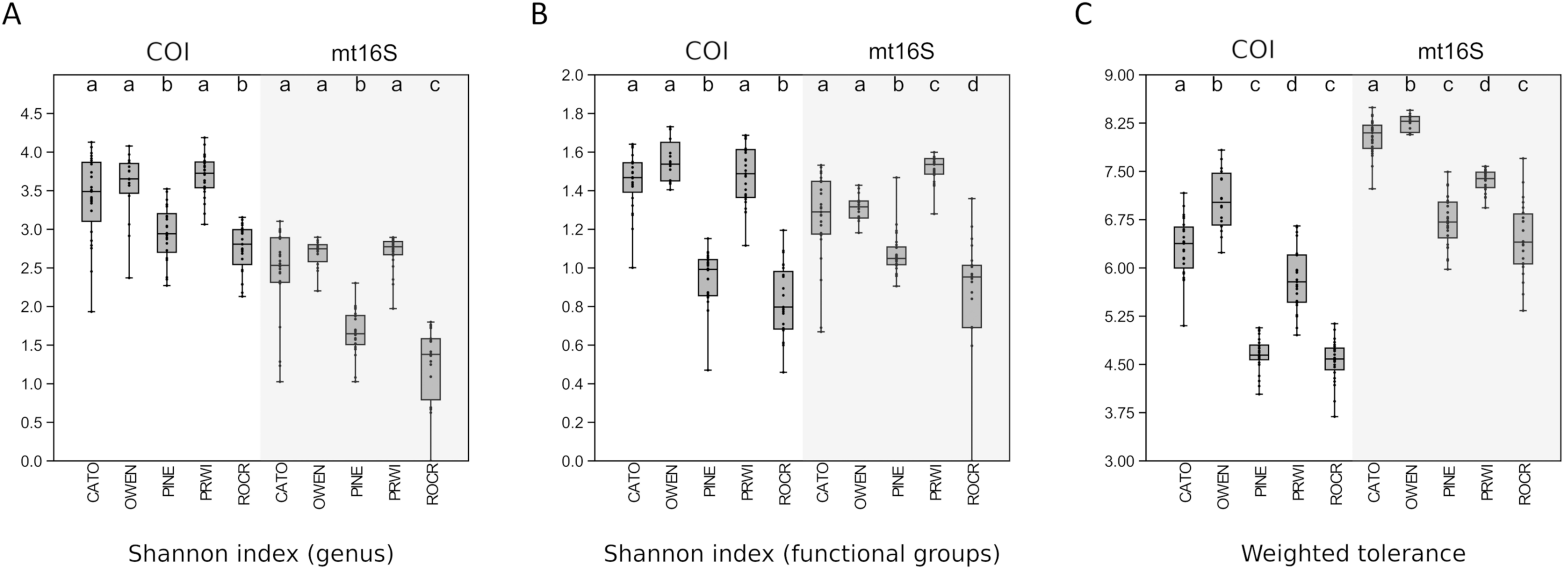
Boxplots of quartiles for three environmental DNA (eDNA) diversity metrics obtained by repeated sampling of five reaches. A significant difference of medians was found by Kruskal-Wallis test for all three metrics at both loci; letters indicate significant pairwise differences within each locus based on subsequent Mann-Whitney U tests. (A) Shannon index of genus proportions in transformed data. (B) Shannon index of functional group proportions in transformed data. (C) Sample tolerance score weighted by genus proportions in transformed data. COI, cytochrome c oxidase 1; mt16S, mitochondrial 16S.

Among-reach variation in arthropod summary metrics is summarized in Fig. 3. Values shown are genus-level Shannon diversity, functional-group Shannon diversity, and weighted tolerance score. The COI locus generated higher genus-level Shannon index values than mt16S, consistent with the paired sampling results. At both loci, significantly lower diversity metrics were obtained for PINE and ROCR compared with PRWI. Mean Shannon index values for arthropod genera at the physiographically distinct CATO and OWEN sites were similar to each other and to PRWI. Shannon index values for functional groups also strongly differentiated ROCR and PINE samples from PRWI, whereas CATO and OWEN scored very similarly to PRWI for COI taxa but had lower functional diversity than PRWI for mt16S taxa. Weighted tolerance scores were generally higher at mt16S than COI, as was seen with paired sampling and consistent with the compositional biases highlighted by Figs. 2 and S6. CATO and particularly OWEN had higher weighted tolerance scores at both loci relative to PRWI. Separating scores by spring 2020 *vs*. summer 2019 sampling indicates a modest effect of season overall (Fig. S12), although less sampling was performed in spring.

**Figure 4:**
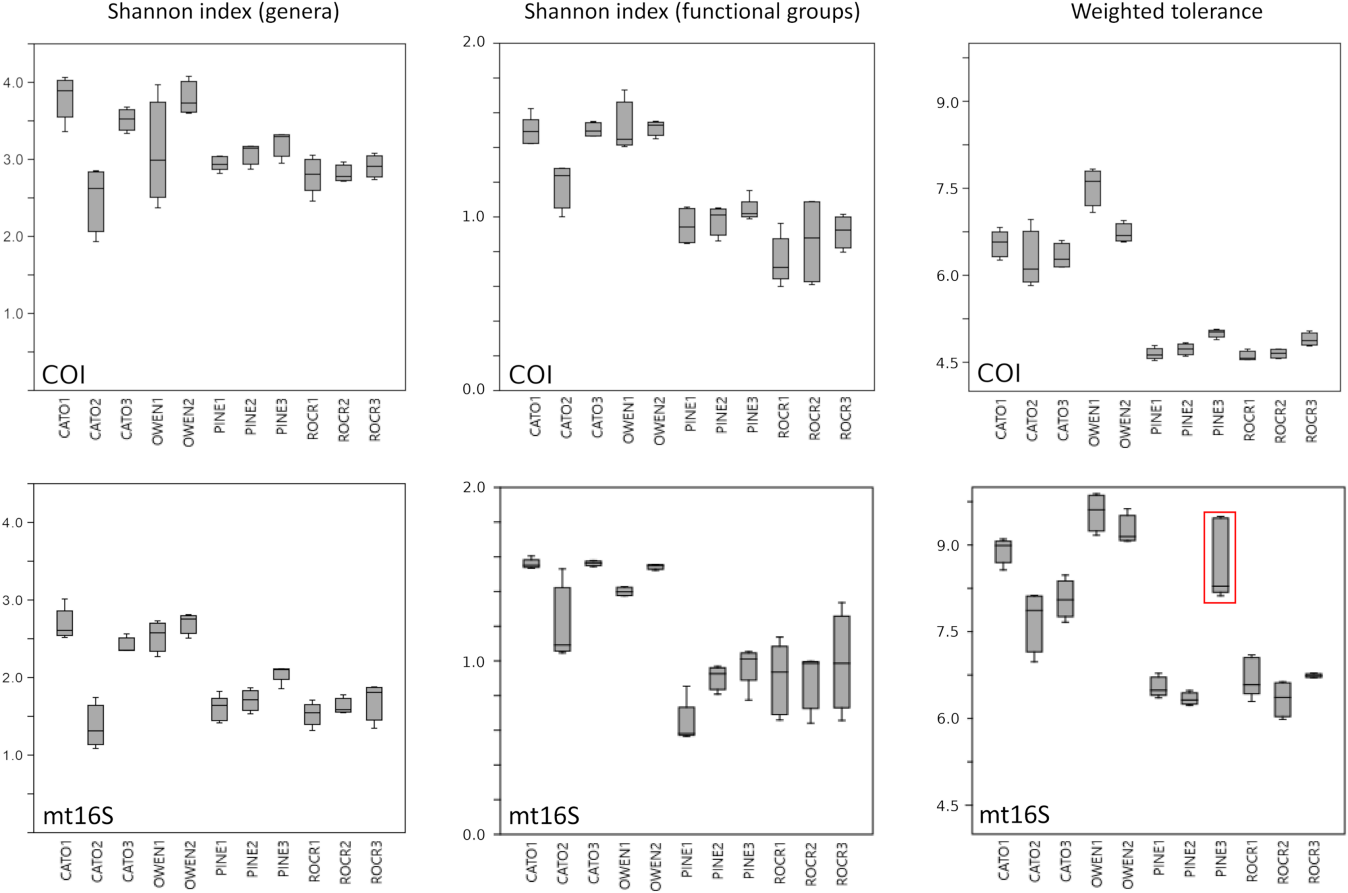
Within-reach variation in three environmental DNA (eDNA) diversity metrics, from replicate samples obtained for the month of September only (*n* = 3–5 per site). Boxplots indicate quartile values for each site (not fully defined for *n* < 4). Filter replicates were not obtained for Prince William Forest (PRWI) sites in September because a high level of suspended material prevented the use of the 0.2 micron filter. The red box highlights an unusually high weighted-tolerance score derived from mt16S taxa at PINE3. COI, cytochrome c oxidase 1; mt16S, mitochondrial 16S.

**Figure 5:**
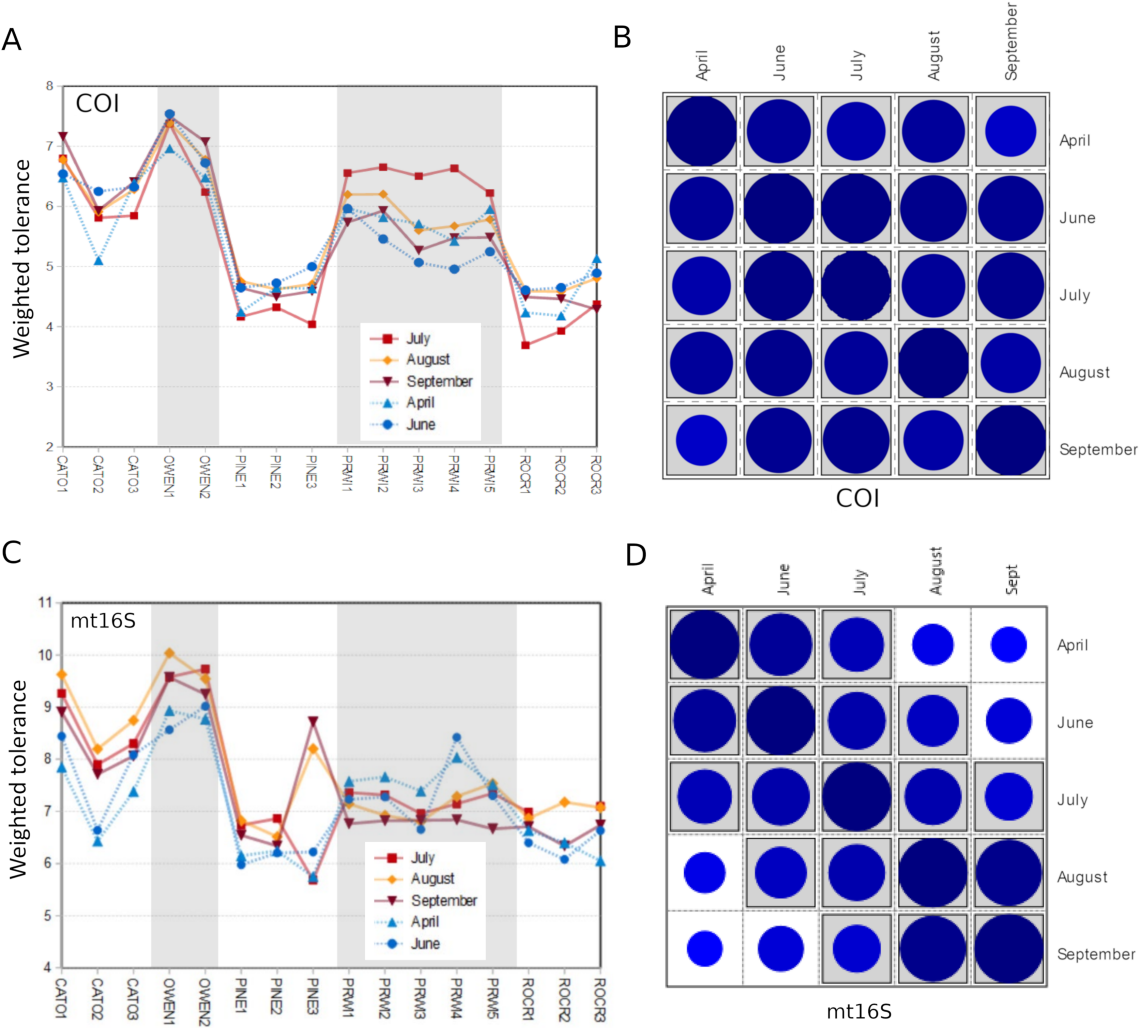
Seasonal comparison of weighted tolerance scores by site within reach. September values are the average of all replicates for that month (other months were not replicated at the site level). (A) Trends by site and reach for COI weighted tolerance scores. (B) Pairwise Pearson correlation matrix for the values in panel A (*n* = 16 sites). The magnitude of each correlation coefficient is proportional to the size of the circle for all cross-month comparisons (range = 0.72–0.98 for all cross-month comparisons). Correlation coefficients significantly greater than zero after Bonferroni adjustment for multiple tests are shaded gray. (C) Trends by site and reach for mt16S weighted tolerance scores. (D) Pairwise Pearson correlation matrix for the values in panel C (*n* = 16 sites). The magnitude of each correlation coefficient is proportional to the size of the circle (range = 0.51–0.96 for all cross-month comparisons). Correlation coefficients significantly greater than zero after Bonferroni adjustment for multiple tests are shaded gray. COI, cytochrome c oxidase 1; mt16S, mitochondrial 16S. See Fig. 1 for a map of site locations.

We assessed within-reach variation in two ways. First, we used the additional biological replicates taken at four of the five watersheds for technical validation during September 2019 to assess site-level variation within that month only. Second, we compared the consistency of point estimates for sites within reaches across all five survey months (for which we averaged the replicated September values). Within-reach comparisons for September sampling were broadly similar between loci and across metrics, and little consistent differentiation was seen among sites within reaches other than at CATO2 (Fig. 4). CATO2 scored more poorly than other CATO sites in five of the six panels of Fig. 4, with the exception being COI tolerance scores. PINE3 was a strong outlier at mt16S in September, which by inspection (File S10) was influenced by high proportions of crayfish (genus *Cambarus*) not detected at either locus at other PINE sites or at PINE3 in other months. This observation highlights the potential danger of transiently detected taxa influencing metrics derived from single sampling events. The relative ranking of sites by tolerance score was more consistent over time with COI than mt16S (Fig. 5). The mt16S locus more consistently identified the CATO2 site as potentially degraded relative to other CATO sites (see Materials and Methods), but the visibly sedimented PRWI1 site did not score differently from other PRWI sites at either locus. Interestingly, eDNA metrics consistently show positive trends from PINE1 to PINE3 (Fig. 4), which corresponds to increasing distance from the urban boundary of Rock Creek Park (Fig. 1D).

**Figure 6:**
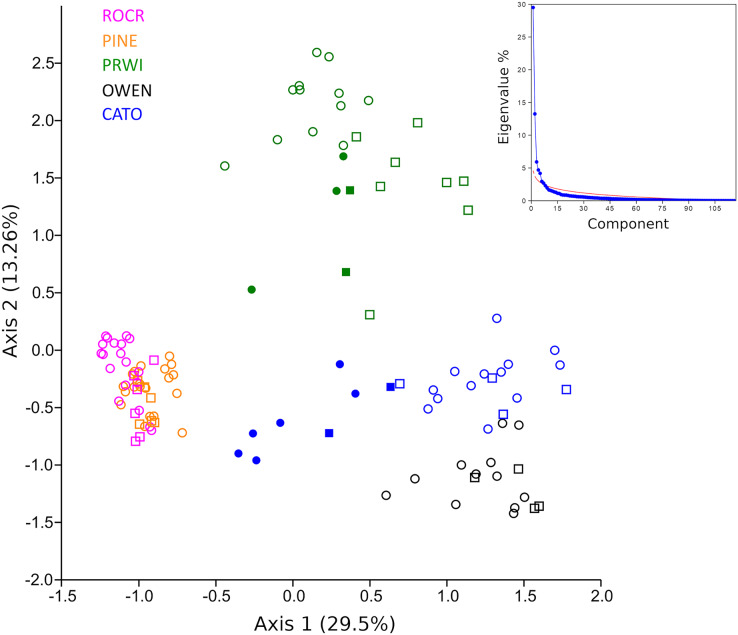
Principle components analysis (PCA) of transformed compositions of arthropod genera detected with repeated environmental DNA (eDNA) sampling in five watersheds. Circles represent 2019 samples and squares represent 2020 samples. Filled green points represent PRWI1 samples and filled blue points represent CATO2 samples (see text for details). The same genus detected at both loci was treated as two independent variables. The proportion of the total variation explained by each axis is given in parentheses. A scree plot comparing the variation explained by each axis (eigenvalue percentage) relative to a null ‘broken stick’ model is shown in the inset, indicating that the plotted axes explain more variation than expected by chance.

A PCA analysis of combined mt16S and COI compositions was performed after excluding taxa present in fewer than three samples. Samples showed strong clustering by stream reach when plotted on the top two axes of variation (Fig. 6). These axes explain significantly more variation (42.8% of the total) than expected under a ‘broken stick’ null model (Fig. 6 inset). The first axis indicates strong differentiation between urban stream reaches (PINE and ROCR) and nonurban stream reaches (PRWI, CATO, and OWEN), consistent with previous studies that have shown a dominant effect of urban land use on biological indices (Vølstad et al., 2003; Goetz & Fiske, 2008; Utz, Hilderbrand & Boward, 2009; Stranko, Hilderbrand & Palmer, 2012; Lookingbill et al., 2014). The second PCA axis is consistent with physiographic differences between the warmer, low-elevation PRWI sites and higher-elevation, higher-gradient CATO and OWEN sites. All CATO2 samples (filled blue symbols) had lower values on PCA1 than other CATO samples. It should also be noted that CATO2 was less than 1 km downstream of a small lake (Fig. 1), which could have impacted the biota detected. PRWI1 samples tended to have lower PCA1 scores than other PRWI samples, but not consistently so, although a single PRWI1 sample fell close to the PINE and ROCR clusters. We conclude that combining COI and mt16S compositions in a single PCA analysis revealed patterns of variation among reaches strongly associated with stream environment, and also indicated within-reach variation consistent with environmental degradation directly observed or previously reported at PRWI1 and CATO2 (see Materials and Methods).

**Figure 7:**
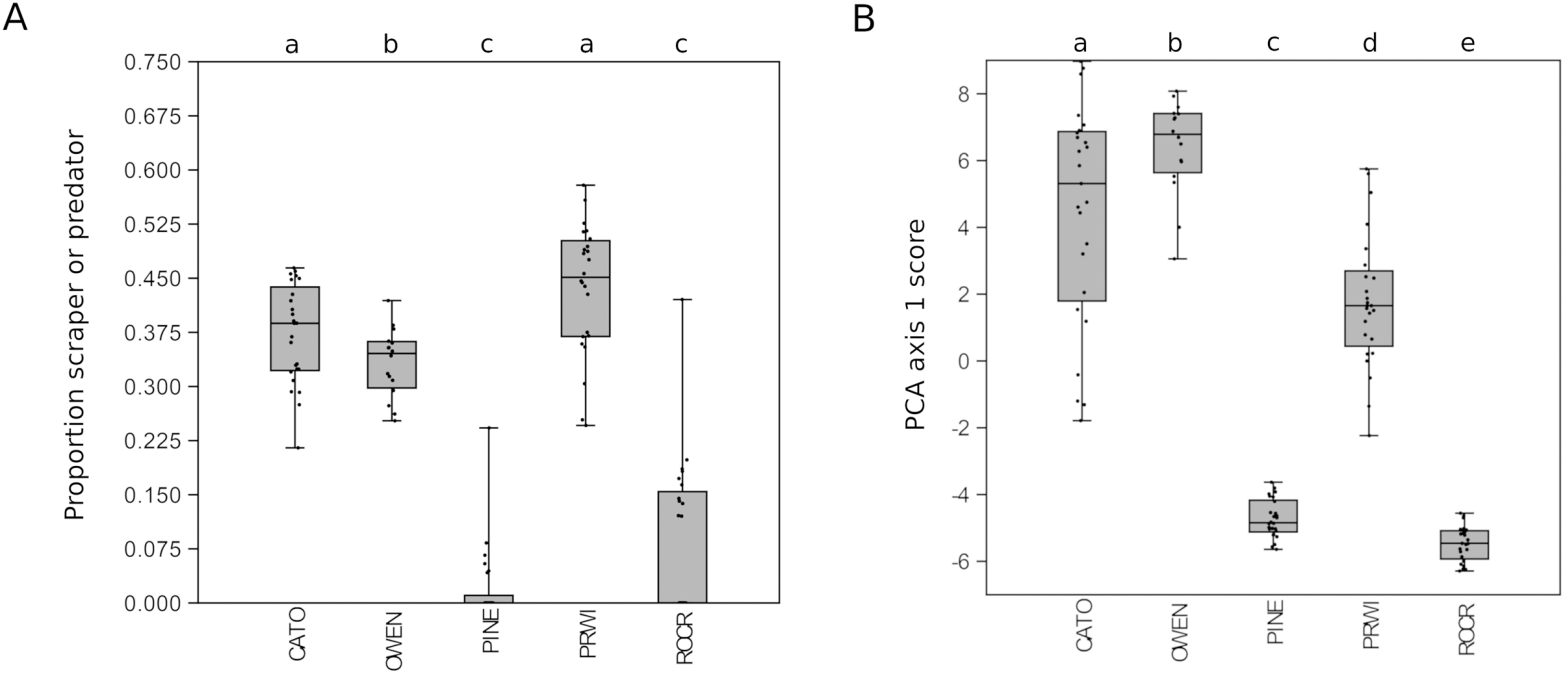
Potential metrics of arthropod diversity that strongly differentiated reaches. Boxplots represent the quartiles of the distribution and points represent values for individual samples, jittered for legibility. A significant difference of medians was found by Kruskal-Wallis test for both metrics; letters indicate significant pairwise differences within each locus based on subsequent Mann-Whitney U tests. (A) Proportion of mt16S taxa classified as scraper or predator relative to all functional groups combined. (B) Scores obtained for samples from the first axis of the principal components analysis (PCA) illustrated in Fig. 6. mt16S, mitochondrial 16S.

Given the strong patterns of differentiation between ‘poor’ and ‘good’ sites evident in functional-group proportions (Fig. S5) and the two-locus PCA analysis (Fig. 6), we propose that these variables may be particularly efficient eDNA metrics for future testing on a wider range of independently characterized sites. Boxplots of the combined proportion of scrapers and predators among mt16S taxa and the locus- and genus-specific PCA1 loadings confirmed strong differentiation among stream reaches by these metrics (Fig. 7).

**Figure 8:**
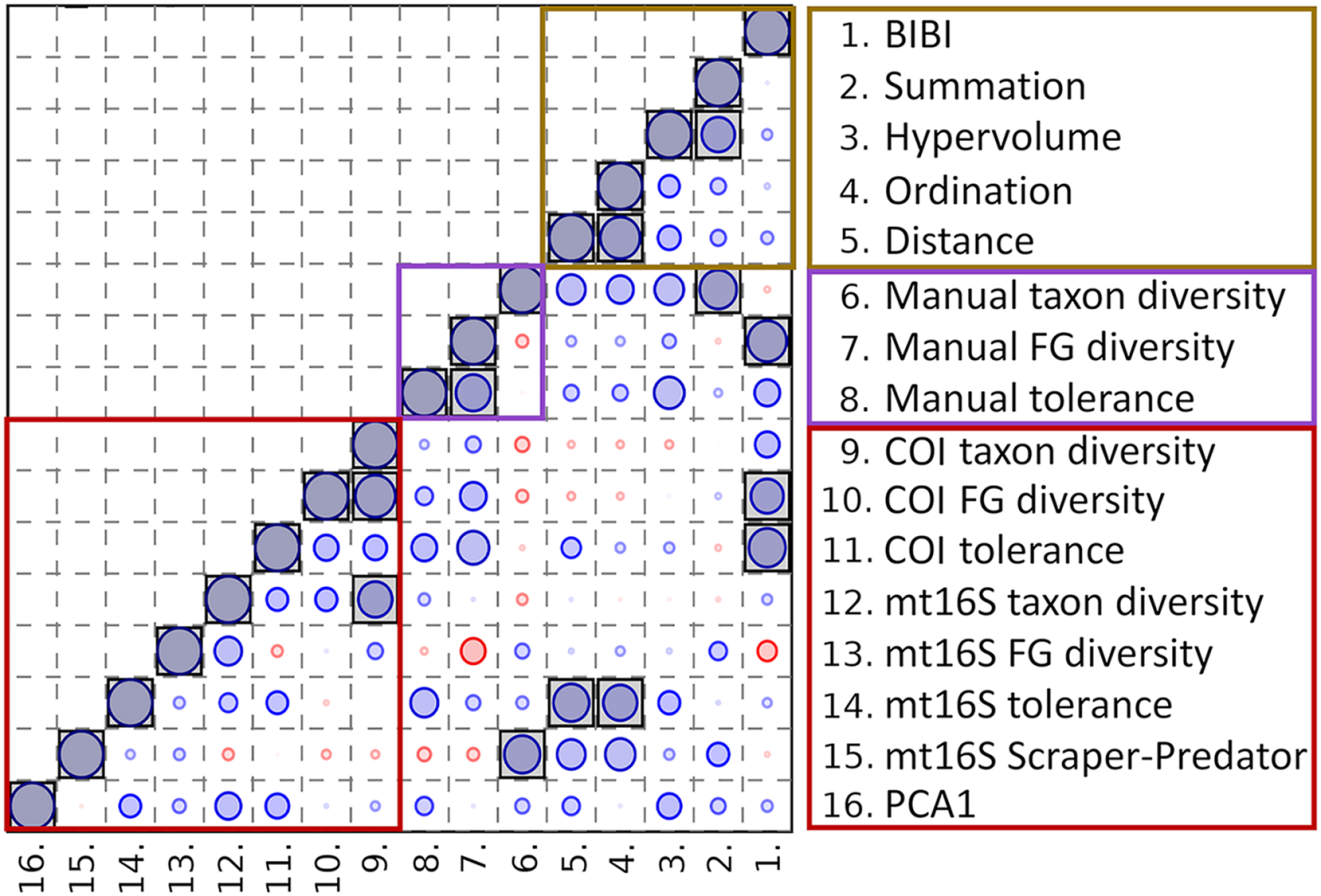
Spearman’s rank correlations between five environmental quality metrics from other studies that integrate multiple variables (see text for details) and eleven metrics derived from manual and environmental DNA (eDNA) sampling in this study. Single values for each metric were derived for eight units of the National Park Service (NPS) as described in the text. The size of the circle in each matrix cell is proportional to the pairwise correlation coefficient, with blue circles indicating the coefficient is positive and red circles indicating negative coefficients. Shaded cells indicate the pairwise comparison is significantly different from zero (unadjusted *P* < 0.05). No false-discovery correction is applied because the tests are not independent, therefore significance levels should be interpreted with caution. BIBI, Benthic invertebrate biological index; FG, functional group; PCA1, first principal components axis. COI, cytochrome c oxidase 1; mt16S, mitochondrial 16S.

Averaging eDNA metrics and other independent biomonitoring scores at the park level suggests that these variables are often complementary rather than redundant (Fig. 8). Reported BIBI scores are most similar to COI diversity metrics and manually derived functional-group diversity. The metrics of Lookingbill et al. (2014) fell into two clusters: the hypervolume and summation scores were most similar to manually derived taxon diversity, whereas the ordination and distance scores were significantly correlated with mt16S tolerance scores (see File S12 for exact test results).

## Discussion

### Study results

In this study, we evaluated arthropod metabarcoding of water samples for stream biomonitoring, focusing on the Potomac River watershed of the U.S. mid-Atlantic region. We used paired sampling to directly compare eDNA compositions with standard manual surveys, and we used repeated sampling to assess the degree to which management areas could be differentiated using metrics analogous to those incorporated into standard biological indices. Genus-level arthropod compositions revealed by COI and mt16S metabarcoding generally had low levels of overlap with paired manual samples at the site level (Fig. S4), and each barcode detected taxonomic and functional groups in different proportions (Fig. 2). Of the two loci, COI compositions were more similar to manual sampling in terms of site-level overlap and detecting those taxa most prevalent in manual samples (Fig. S2). Importantly, the two loci recovered very different compositions of Chironomidae and EPT taxa (Fig. 2), which strongly influenced the magnitude of weighted tolerance scores at each locus but not the relative rankings of reaches (Fig. 3C). On the other hand, a higher proportion of mt16S taxa were found at a majority of the paired-sampling sites (Fig. S2), mt16S compositions more strongly differentiated sites with repeated sampling (Fig. S5), and mt16S compositions had higher technical replicability (Fig. S10).

The low site-level overlap between eDNA and manual surveys is perhaps unsurprising since waterborne eDNA flows through a study site from potentially diverse sources and co-amplifying taxa can strongly limit the realized sequencing effort for arthropod BIs, whereas the manual method is specifically designed to capture these taxa. Overlap was also low in the paired manual and aquatic eDNA sampling reported by Hajibabaei et al. (2019a), Gleason et al. (2021), and Pelletier et al. (2022), all of which used various COI primer sets. Hajibabaei et al. (2019a) and Gleason et al. (2021) bulked manually collected samples for metabarcoding rather than used eDNA metabarcoding, whereas Pelletier et al. (2022) compared aquatic and soil eDNA to insects identified from emergence traps. Stochastic variation in the manual sampling process is also a likely contributor to low overlap: for example, the manual surveys of Mueller (2016) identified more unique taxa per site than were shared with prior manual surveys at approximately the same locations.

Despite the low overlap with manual observations at the taxon level, aquatic eDNA provided useful information for metric-based BI biomonitoring, which is comparable to the findings of Brantschen et al. (2021). In paired sampling, metrics derived from manual sampling (Brillouin’s index of total diversity, functional group diversity, and weighted tolerance) were all rank correlated with one or both eDNA tolerance scores. Functional diversity at COI was in fact the only metric that was significantly rank correlated with past BIBI scores at the site level. When values for sites were averaged within parks and also compared to scores generated by Lookingbill et al. (2014) using four methods, tolerance and functional diversity generated by manual and COI surveys were collectively similar by rank to averaged BIBI scores, whereas mt16S tolerance scores were similar by rank to the ordination and distance methods of those authors. Thus, even without substantial replication, metrics obtained from eDNA were consistent with at least some other independent measures, and moreover, the two loci appeared to give complementary rather than redundant information.

In repeated sampling, eDNA-derived metrics at the reach level were consistent between loci and statistically differentiated reaches consistent with expectation from prior resource assessments (Figs. 3 and 7). Individual sites within a reach suspected of being impaired based on prior knowledge or observed factors (*i.e.*, CATO2 and PRWI1) also showed deviations from other sites within the same reach by PCA analysis (Fig. 6), but only CATO2 was differentiated by tolerance and diversity metrics (Figs. 4 and 5). While these results are promising for the application of tolerance scores weighted by relative taxon proportions in eDNA data, we do not claim that our formulation of these metrics is preferrable to another, only that it allows an intuitive comparison of compositions derived by different methods.

We found even greater differences among reaches when focusing on the proportion of scraper and predator classifications, as these groups drive much of the difference seen among sites (Figs. S5 and 7). However, our functional-group analysis was limited to the majority-rule classification of each genus, which could introduce bias and reduce sensitivity relative to a species-level application. Future efforts could attempt to split the weighting factor derived from taxon proportions among multiple genus-level functional groups, for example. Nonetheless, the fact that mt16S-weighted functional diversity and tolerance scores showed consistent reach-level patterns provides support that these classification schemes are informative and usefully applied at the genus level.

On the other hand, *de novo* derived relationships between composition and habitat condition may ultimately allow more taxa to be included in eDNA metrics, and with fewer assumptions. For example, we found that ordination of samples with both mt16S and COI compositions were consistent with urban land use and physiography as major drivers (Fig. 6), as previously shown for manually surveyed BIs (Stranko, Hilderbrand & Palmer, 2012; Utz, Hilderbrand & Boward, 2009; Lookingbill et al., 2014), and that PCA axis 1 scores differentiated stream reaches as well as any other metric (Fig. 7). Notably, scores generated for paired-sampling sites using the PCA loadings of axis 1 did not correlate significantly with any other metric, which may reflect the fact that it merges information from both loci. Broader use of this approach would require further benchmarking, however, by sampling a range of site qualities stratified by physiography, and could be confounded if taxonomic signals of environmental quality are strongly nonlinear (*e.g*., Utz, Hilderbrand & Boward, 2009; Dodds et al., 2010). Intriguingly, the proportion of assignable arthropod sequences at the two barcode loci differentiated reaches comparably to other eDNA metrics and may reflect the relative biomass of benthic invertebrates in streams. However, the dependence of arthropod read rate on the proportions of other clades (*e.g.*, vertebrates or annelids) in sequence data might limit its applicability as an environmental quality metric.

### Limitations and caveats

We used log-ratio scaled relative proportions of arthropod genera in sequence data to weight genus-level tolerance and functional-group classifications specifically developed for the region (Norris & Sanders, 2009), as these are the analogous formulations of commonly used manual BI metrics. This should not be interpreted as an assumption of, or requirement for, any actual correspondence between taxon proportions in eDNA sequences and the environment. The empirically demonstrated similarity between eDNA metrics formulated in this way and other measures of environmental quality imply only that such metrics contain potentially useful information about environmental quality. Ultimately, we seek eDNA-derived metrics that usefully relate to independent environmental variables (*e.g.*, Bista et al., 2017; Mächler et al., 2019; Robinson et al., 2022), regardless of whether taxon proportions in eDNA sequences resemble the counts of organisms in any defined microenvironment that we can directly observe. We note that proportions-based Shannon diversity is widely used in metabarcoding work (*e.g*., Andersen et al., 2012), and some studies have found broadly positive relationships between censused taxa and their proportions in eDNA data (*e.g*., Carew et al., 2013; Kraaijeveld et al., 2015; Elbrecht et al., 2017; Cornman, McKenna & Fike, 2021), but also that numerous counterexamples exist (*e.g*., Gleason et al., 2021) and we do not attempt to review the many relevant studies here (see Pawlowski et al., 2018 for further discussion). It seems reasonable to hypothesize that sample-level summary metrics weighted by the proportions in sequence data of dozens of genera may gain more information than is lost or distorted by amplification and assignment biases, particularly since no test of differential abundance of a specific taxon is being performed, but it would be naïve to forego empirical validation or further optimization. Nonetheless, with the current data, we can at least examine how proportional weighting of tolerance scores compares with equal weighting, by recalculating tolerance scores as unweighted averages across all genera detected above some minimum threshold (here we use 1% of the total). For the Spearman correlation of single estimates of eDNA metrics with manual and external metrics (Fig. S13), we observe that equal weighting substantially degrades the correlation with mt16S-derived tolerance scores but has minimal impact on COI-derived tolerance scores. For the statistical comparison of median tolerance scores among reaches (Fig. S14), weighted and unweighted tolerance scores both yield significant Kruskal-Wallis tests and very similar pairwise relationships, however the strength of the association is weaker with unweighted tolerance scores (as indicated by the magnitude of the test statistic H). We conclude that weighting tolerance scores by taxon proportions in transformed compositional data strengthens, but does not distort, the association between eDNA metrics and independent quality metrics in this study.

The utility of eDNA metrics ultimately hinges on protocols that are both sufficient and efficient in detecting arthropod taxa with acceptable accuracy. We examined a number of technical factors in this study but additional sampling variables could be explored. For example, seasonality is an important consideration in manual BI monitoring and guidelines often recommend seasonal breadth and consistency of sampling (Gibson et al., 1996; Barbour et al., 1999). In repeated sampling, we obtained similar reach-level distributions of eDNA metrics when plotting samples from summer 2019 separate from those obtained in spring 2020 (Fig. S12). Note that seasonal variation in summary metrics is a separate issue from seasonal variation in the underlying taxa (*e.g*., Jensen et al., 2021) from which those metrics are derived. However, some seasonal variation was nonetheless evident for mt16S, and we are not able to address seasonal variation outside of our sampling period or indeed whether seasonal variation is consistent year-to-year. Additional research is needed to determine the extent to which arthropod eDNA metrics are sensitive to variation at this level.

An unresolved question is whether a single-locus approach would be both adequate for management questions and more cost-effective. In this study, COI and mt16S metrics gave very similar results at the reach level (Fig. 3) and indeed those metrics appear well-correlated across all samples in the repeated sampling stage (Fig. S15). However, the apparent correlation between metrics from the two loci could be exaggerated by the strong bimodality of mt16S compositions; that is, the first two panels in Fig. S15 could potentially be interpreted as containing two distinct distributions of points, within which the Pearson correlation coefficient would be much lower. Sampling more sites of intermediate quality would help clarify the extent to which these two loci give complementary or redundant information. In the absence of any other constraint, using multiple loci would certainly yield greater total and trophic diversity (*e.g.*, Robinson et al., 2022), increase confidence in detections (*e.g*., Alberdi et al., 2018), and inform other management questions such as fish diversity. On the other hand, sequencing multiple loci also entails greater overall cost and processing complexity and dilutes the per-sample sequencing effort, with potentially important effects on the saturation and replicability of compositions.

If multilocus approaches are used, an additional question is whether and how those data should be combined. For example, we treated the same taxon identified at COI and mt16S as independent entities in our ordination analysis because taxon counts at the two loci are approximately uncoupled by the nature of compositional data. That is, the inferred proportion of a taxon at a locus is strongly constrained by all of the other taxa detected with the same primers, rather than by the absolute abundance of the organisms themselves in the environment. Regardless, BI scoring schemes have long used binning methods to integrate multiple metrics that are intrinsically correlated, such as the number of EPT taxa detected as well as their proportion in a sample (*e.g*., Southerland et al., 2007). Treating the proportions of the same taxon in sequence data derived from different loci as distinct components of an eDNA-based biological index would be consistent with this practice.

## Conclusions

The two metabarcoding loci gave complementary rather than redundant information about environmental quality in these environments, due to distinctive taxonomic biases, but were largely concordant in terms of the median values of summary metrics within reaches. While CATO2 scored lower than other sites in the same reach, consistent with some independent data of environmental degradation, that result may also have been influenced by an upstream artificial lake and more work is needed to clarify the spatial resolution of these methods within a reach. Our approach made extensive use of functional data aggregated for regional species by previous researchers, which may be lacking for other regions.

### Future Directions

While we recovered DNA from the water column using a standard filtration approach, other eDNA sampling methods may yield complementary or superior information. One alternative approach is autosampling (*e.g*., Scholin et al., 2017; Cornman et al., 2018; Sepulveda et al., 2020) or passive sampling (Bessey et al., 2021). A second alternative approach is metabarcoding of bulked samples collected by traditional methods, which can yield higher diversity than water samples, particularly more EPT diversity (Hajibabaei et al., 2019a). This approach retains the manual effort of sampling benthic habitats but foregoes the taxonomic binning of individual specimens.

As reference databases improve in representation and genetic concordance, taxonomic performance of metabarcoding workflows should also continue to improve. While the mt16S barcode is shorter than the COI barcode, the relative information content of the two loci depends on the magnitude of the barcode ‘gap’ (Puillandre et al., 2012) and may not be directly related to length. For example, first and second codon positions are strongly constrained in COI gene sequences whereas third position variation may be homoplasious. In contrast, the secondary structure of mt16S generates a complex covariance pattern rather than codon-structured variation (Zuker, 2000). Our results suggest that differences in taxonomic resolution may be minor when both loci are comparably represented in a reference database, similar to the results of Elbrecht & Leese (2017). Alternatives to genus-level aggregations could also be explored. For example, binning poorly differentiated species at hierarchical taxonomic ranks is a default procedure due to its simplicity and familiarity, yet binning based on other operational criteria such as assignment error rate (Cornman, McKenna & Fike, 2021) could increase the sensitivity of derived metrics by reducing the number of taxa over which trait values are averaged.

Evidence that eDNA metrics actually relate to environmental quality *per se* remains limited and indirect. In this study, single eDNA sampling events correlated well with other metrics generated *via* more intensive methods (Fig. 8), but the number of park units for which all independent metrics were available was relatively small (*n* = 8) and these metrics were generated from surveys separated by more than a decade. Furthermore, sampling sites within parks were not always the same across the different monitoring studies and we resorted to simple arithmetic averaging to obtain park-level values for our data. Additionally, our results from repeated sampling confirm the potential bias from unrepresentative variation (Fig. 4) and highlight the limitations of comparisons based on few sampling events. It would therefore be valuable to perform additional repeated sampling at already well-characterized sites that represent a range of environmental qualities, stratified by physiography and urban land use, for example using the MBSS survey results (Klauda et al., 1998) as a guide.

## Supplemental Information

10.7717/peerj.15163/supp-1 Supplemental Information 1 Paired-sampling site metadata and matched Benthic Invertebrate Biological Index scores reported in National Park Service resource assessments.

10.7717/peerj.15163/supp-2 Supplemental Information 2 Collection and sequencing metadata for repeated sampling.

10.7717/peerj.15163/supp-3 Supplemental Information 3 Taxon list extracted from taxonomic sources, with corresponding NCBI taxonomy identifiers by which NCBI sequence accessions were filtered.

10.7717/peerj.15163/supp-4 Supplemental Information 4 Formatted cox1 database used in the analysis.

10.7717/peerj.15163/supp-5 Supplemental Information 5 Formatted mt16S database used in the analysis.

10.7717/peerj.15163/supp-6 Supplemental Information 6 Supplementary methods: amplification bias and taxonomic validation.

10.7717/peerj.15163/supp-7 Supplemental Information 7 Taxonomic assignments obtained using the Sintax reference database of voucher mt16S sequences obtained from manually classified benthic-invertebrate specimens.

10.7717/peerj.15163/supp-8 Supplemental Information 8 Neighbor-joining dendrograms of three mt16S voucher sequences and top-matching accessions in the NCBI nucleotide database identified by homology search. The three search queries were incorrectly assigned at the genus level and the resulting phylogenies demonstrate discordance of NCBI reference sequences with assigned taxonomy. Trees were generated using a gamma rate distribution as described in the text. Bootstrap values are shown based on 1,000 resampled replicates, but these are shown for information only and do not imply that these gene trees are sufficient evidence of organismal phylogeny. (A) *Calopteryx* (marked with red circles) and related genera (B) *Faxonius* (marked with blue circles) and related genera (C) *Stenonema* (marked with green circles) and related genera. mt16S, mitochondrial 16S ribosomal RNA gene.

10.7717/peerj.15163/supp-9 Supplemental Information 9 Raw counts and transformed compositions for paired sampling.

10.7717/peerj.15163/supp-10 Supplemental Information 10 Raw counts and transformed compositions for repeated sampling.

10.7717/peerj.15163/supp-11 Supplemental Information 11 Example of a log-ratio transformation of counts prior to analysis.

10.7717/peerj.15163/supp-12 Supplemental Information 12 Input data and output results of statistical analyses reported in the main text.

10.7717/peerj.15163/supp-13 Supplemental Information 13 Arthropod sequence in negative controls and mocks is consistent with a crosstalk rate of 1 in 10,000. (A) Arthropod sequence in pooled field negative controls and laboratory extraction controls. Points indicate the number of reads of a given taxon summed across all biological samples *vs*. each negative control pool (all taxonomic ranks are shown). (B) Arthropod sequence in mock samples of known input composition. Green points indicate the number of reads for taxonomic assignments that are consistent with inputs, whereas black points are inferred to be crosstalk as they scale with total counts of a taxon at approximately 1 in 10,000. Red points are potential contaminants, in that they are visually elevated above the crosstalk distribution. COI, cytochrome c oxidase 1; mt16S, mitochondrial 16S.

10.7717/peerj.15163/supp-14 Supplemental Information 14 Comparison of the prevalence of taxa detected by manual *vs*. environmental DNA (eDNA) sampling across thirteen sites of paired eDNA and manual sampling. COI, cytochrome c oxidase 1; mt16S, mitochondrial 16S.

10.7717/peerj.15163/supp-15 Supplemental Information 15 Rarefaction curves plotted with a step size of 100. (A) Paired sampling with individual sites labeled. (B) Repeated sampling with individual samples unlabeled for clarity. The horizontal range is truncated at 5,000 sequence counts to facilitate a scale-matched comparison, since more reads are obtained at the mt16S locus on average. COI, cytochrome c oxidase 1; mt16S, mitochondrial 16S.

10.7717/peerj.15163/supp-16 Supplemental Information 16 The total number of taxa detected manually at each site at a 1% threshold of total sample counts, compared to the number also detected by environmental DNA (eDNA) at either or both loci. COI, cytochrome c oxidase 1; mt16S, mito.

10.7717/peerj.15163/supp-17 Supplemental Information 17 Key taxonomic and functional groups commonly used in biological indexes are differentially recovered by the two barcode loci and are more spatially structured at mt16S in repeated sampling. Reaches are listed in alphabetical order, but separately for summer 2019 and spring 2020 samples. The proportions of each group are relative, summing to 100% after excluding taxa assigned above the genus level (family level for Chironomidae) in the top panel or lacking a functional-group classification in the bottom panel. (A) Proportion of functional groups at COI. (B) Proportion of functional groups at mt16S. (C) Proportions of common arthropod indicator clades at COI. (D) Proportions of common arthropod indicator clades at mt16S. COI, cytochrome c oxidase 1; mt16S, mitochondrial 16S.

10.7717/peerj.15163/supp-18 Supplemental Information 18 Correlation matrix between average benthic invertebrate biological index (BIBI) scores reported by the National Park Service at a site and metrics derived from paired environmental DNA (eDNA) and manual sampling in this study. The size of the circle in each cell is proportional to the magnitude of the pairwise Spearman’s rank correlation coefficient, with blue circles indicating a positive correlation and red cells indicating a negative correlation coefficient. Gray-shaded cells indicate pairwise correlation coefficients that are significantly different than zero. FG, functional group. See File S12 for exact test results. COI, cytochrome c oxidase 1; mt16S, mitochondrial 16S.

10.7717/peerj.15163/supp-19 Supplemental Information 19 DNA yield from single filter extractions as a function of reach, year, and realized filtrate volume.

10.7717/peerj.15163/supp-20 Supplemental Information 20 Average DNA yield by filter type and reach. The three filter types tested were 0.2 micron cellulose nitrate (0.2 CN), 0.45 micron cellulose nitrate (0.45 CN), and 0.8 micron mixed cellulose esters (0.8 MCE). Lines indicate standard deviations for each reach-filter combination. The 0.2 CN and 0.8 MCE filters were not tested at PRWI.

10.7717/peerj.15163/supp-21 Supplemental Information 21 The proportion of sequence reads assigned to an arthropod genus or to family Chironomidae differed among reaches. A significant difference in medians was assessed by Kruskal-Wallis test with letters indicating pairwise differences by Mann-Whitney U test after sequential Bonferroni adjustment of P-values. (A) Rates of arthropod read recovery at COI by reach. (B) Rates of arthropod read recovery at mt16S by reach. (C) Bivariate plot of median arthropod read rate by site within reaches. COI, cytochrome c oxidase 1; mt16S, mitochondrial 16S.

10.7717/peerj.15163/supp-22 Supplemental Information 22 Distribution of Pearson correlation coefficients by replicate type and replicate properties. (A) Pairwise correlations of taxon compositions in strict technical replicates processed with the same protocol (*n* = 8). (B) Pairwise correlations of taxon compositions between technical replicates processed with different kits for removing PCR inhibition (*n* = 11, see text for details). (C) Pairwise correlation of taxon compositions in biological replicates obtained with one of three different filter types (*n* = 25, see text for details). All possible pairwise combinations were included. (D) Pearson correlation coefficients of taxon compositions between strict technical replicates as a function of extracted DNA concentration. (E) Pearson correlation coefficients of taxon compositions between strict technical replicates as a function of sequencing effort, measured as average number of reads assigned to arthropod genera. (F) Pearson correlation coefficients of taxon compositions between strict technical replicates as a function of the total richness of the two replicates. COI, cytochrome c oxidase 1; mt16S, mitochondrial 16S.

10.7717/peerj.15163/supp-23 Supplemental Information 23 Replicate dropout, defined here as the detection of a taxon in only one of two technical replicates, is a function of log-ratio transformed proportions. Each point represents the log-ratio transformed proportions of a taxon in two technical replicates, with all eight technical replicates plotted together. While taxon dropout occurs more frequently at lower values, log-transformed proportions are similar across the observed range of values when detected in both replicates. (A) COI transformed proportions (B) mt16S transformed proportions. COI, cytochrome c oxidase 1; mt16S, mitochondrial 16S.

10.7717/peerj.15163/supp-24 Supplemental Information 24 Seasonal effects on eDNA metrics were modest. 2019 samples were collected in July, August, and September, whereas 2020 samples were collected from April to early June. Boxplots indicate range, mean, and quantiles for each site. Vertical-axis scale is fixed between loci for a given metric to facilitate comparison. (A) Shannon index of COI-detected arthropod genera (B) Shannon index of mt16S-detected arthropod genera (C) Shannon index of COI-detected functional groups (D) Shannon index of mt16S-detected functional groups (E) COI weighted tolerance score (F) mt16S weighted tolerance score.

10.7717/peerj.15163/supp-25 Supplemental Information 25 Weighting tolerance by log-ratio transformed proportions improves the correlation between point estimates and independent quality metrics at mt16S but not COI. For unweighted tolerance, the scores for all genera present at greater than 1% of the sample sum (after log-ratio transformation) were averaged without weighting to obtain the sample-level tolerance score. (A) Spearman pairwise correlation matrix from Fig. S6 with additional columns for unweighted tolerance. (B) Spearman pairwise correlation matrix from Fig. 8 with additional columns for unweighted tolerance. BIBI, benthic invertebrate biological index; Brill, Brillouin’s index; H, Shannon index; HFG, Shannon index of functional groups; T_W_, weighted tolerance; T_U_, unweighted tolerance. COI, cytochrome c oxidase 1; mt16S, mitochondrial 16S.

10.7717/peerj.15163/supp-26 Supplemental Information 26 Weighting environmental DNA (eDNA) tolerance metrics by log-ratio transformed proportions strengthens the statistical differences among reaches in repeated sampling without altering the biological pattern. H is the test statistic of the Kruskal-Wallis test of median score among reaches, and reaches that share a lower-case letter are not significantly different by pairwise Mann-Whitney U test. See File S12 for test details. (A) Distribution of sample-level tolerance scores for COI-detected genera, weighted by transformed proportions. (B) Distribution of sample-level tolerance scores for mt16S-detected genera, weighted by transformed proportions. (C) Distribution of sample-level tolerance scores for COI-detected genera, equally weighted across those genera comprising >0.01 of the total after log-ratio transformation. (D) Distribution of sample-level tolerance scores for mt16S-detected genera, equally weighted across those genera comprising >0.01 of the total after log-ratio transformation. COI, cytochrome c oxidase 1; mt16S, mitochondrial 16S.

10.7717/peerj.15163/supp-27 Supplemental Information 27 Linear regression of environmental DNA (eDNA) metrics derived from mt16S taxa on those derived from COI taxa. COI, cytochrome c oxidase 1; mt16S, mitochondrial 16S.

## Additional Information and Declarations

### Field Study Permissions

The following information was supplied relating to field study approvals (*i.e*., approving body and any reference numbers): Collections were performed under permits CATO-2019-SCI-0009, PRWI-2019-SCI-0010, and ROCR-2019-SCI-0019 of the National Park Service

### Data Availability

The following information was supplied regarding data availability: The sequence data are available at BioProject: PRJNA877240. The arthropod sampling data are available in the thesis at PennState University Libraries: https://etda.libraries.psu.edu/catalog/13548sjm5467. By agency policy, the raw data have also been made available at the USGS’s ScienceBase portal: Cornman, R. S., Aunins, A. W., and Fike, J. 2023, Metabarcode sequencing of aquatic environmental DNA from the Potomac River Watershed, 2015–2020: U.S. Geological Survey data release, https://doi.org/10.5066/P9NNZNVH.
