# Supplementary material for: Assessing arthropod diversity metrics derived from stream environmental DNA: spatiotemporal variation and paired comparisons with manual sampling
Source: PeerJ. 2023 Mar 31;11:e15163. doi: 10.7717/peerj.15163 (PMC10069422; doi:10.7717/peerj.15163)
Supplement: Supplemental Information 6 [file peerj-11-15163-s006.docx]

**Supplemental File S6. Supplementary methods: amplification bias and taxonomic validation.**

*Assessment of primer bias with mock samples*

To test primer performance on regionally representative benthic arthropods, six specimens collected manually at sites in the paired sampling survey and classified to genus were used to generate a mock community. DNA was extracted from single ethanol-preserved specimens of *Acroneuria* (Plecoptera), *Argia* (Odonata), *Baetis* (Ephemeroptera), *Eccoptura* (Plecoptera), *Micrasema* (Trichoptera), *Tricorythodes* (Ephemeroptera). DNA was with the Qiagen DNEasy Blood and Tissue kit per manufacturer’s instructions, with the exceptions that samples were homogenized with a sterile plastic pestle after placement into buffer ATL to facilitate tissue lysis and the final elution volume was decreased to 100 µl. DNA concentration of the extracts was quantified using a Qubit dsDNA High Sensitivity quantification kit with 2 µl of sample. Amplicons of each barcode locus were generated for each specimen in 50 µl reaction volumes containing 0.25 µl of GoTaq DNA polymerase (Promega) at a stock concentration of 5units/µl, 10 µl reaction buffer, 4 µl of 25mM MgCl_2_, 5 µl of forward and reverse primers at 10 µM concentration each, 2 µl template solution, 1 µl dNTPs (10 mM each), and 27.75 µl water. The thermocycling program included an initial denaturing at 95 °C for 3 min, followed by 35 cycles of 95 °C for 1 min, 58 °C (COI) or 60 °C (mt16S) for 1 min, 72 °C for 1 min, and a final extension at 72 °C for five minutes. PCR products were cleaned with a Qiagen QIAquick PCR purification kit following the manufacturer’s instructions and eluted in 50 µl water. Each amplicon was visualized on an Agilent BioAnalyzer 2100 using High Sensitivity reagents to confirm a single peak of the expected size. Concentration of each amplicon was estimated in triplicate with the Qubit dsDNA HS kit and the results averaged. Amplicons were then pooled equivolume by locus to generate inputs for sequencing libraries in triplicate, as described in the main text.

Taxonomic proportions of mock-community members in the resulting sequences were assessed by direct mapping of processed reads with bowtie2 v. 2.4.5 (Langmead and Salzburg 2012) to a set of representative sequences for each genus and locus. No mt16S sequences were available in the nucleotide (nt) database of the National Center for Biotechnology Information (NCBI) for either *Micrasema* or *Tricorythodes*, however. Putative *Tricorythodes* reads could be identified by homology to related genera and were added to the bowtie2 database, yet no *Micrasema*-like sequence was recovered at mt16S. Overall, taxonomic proportions recovered for the mock community were highly similar among replicates but differed consistently from expected proportions based on input DNA concentration of amplicons. *Baetis* was consistently over-represented whereas *Micrasema* and *Tricorythodes* were under-represented or absent. We conclude that the two primer sets performed similarly on templates likely to be present in environmental samples.

**
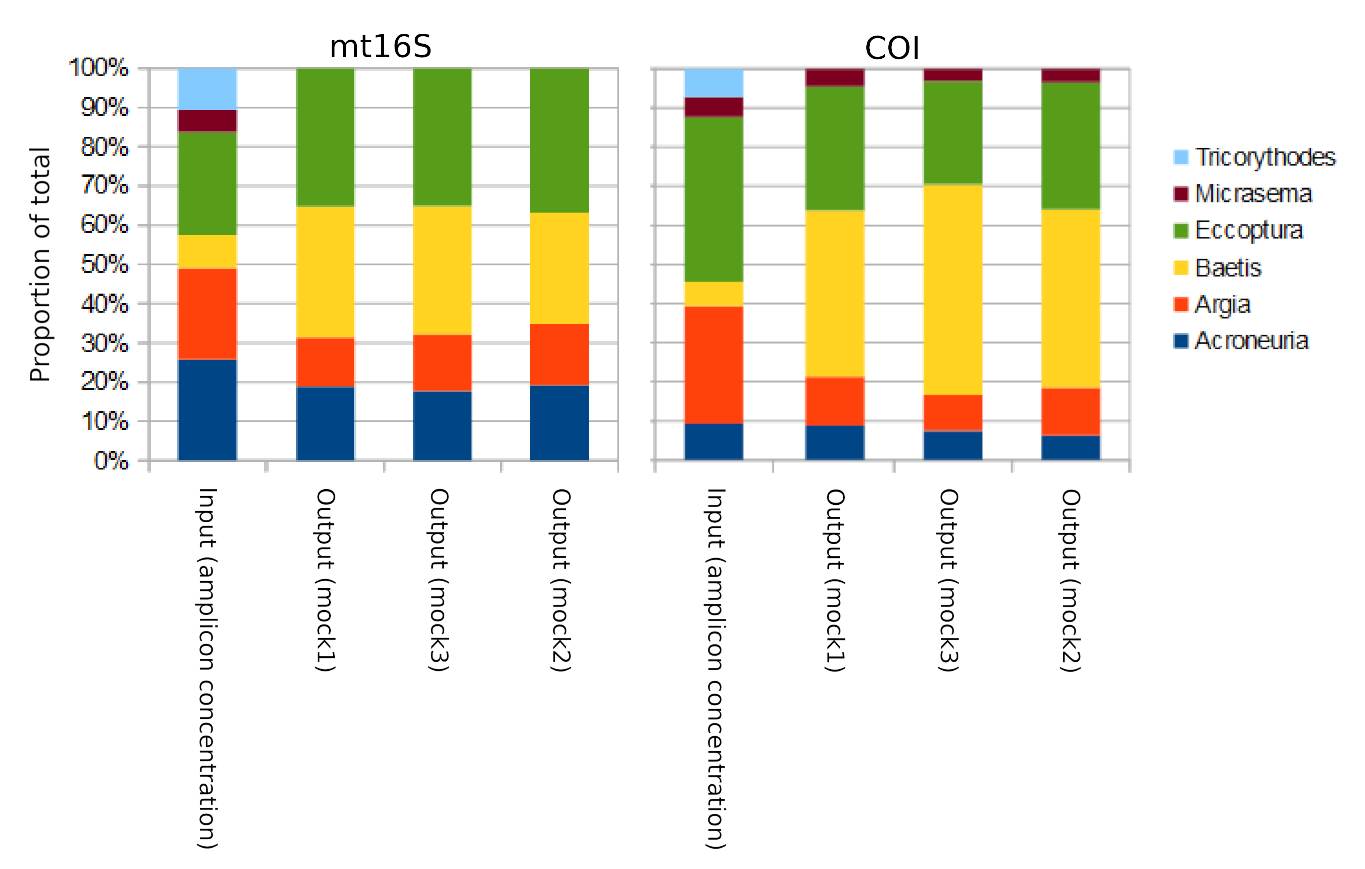
**

**Amplification bias in replicate mock communities of six arthropod taxa.** Expected input proportions are based on relative concentrations of amplicon prior to pooling. Observed output proportions are based on direct mapping of reads to reference sequences for each taxon.

*Validation of taxonomic methods*

The number of unique genus-level taxonomic IDs identified from the taxon lists of Mueller (2016) and the MBSS (Maryland Department of Natural Resources, 2021) was 579. The COI database we generated had 66,635 sequences representing 24,997 unique species IDs within 559 genera. The mt16S database had 1,422 sequences representing 990 unique species IDs within 169 genera. Note that many provisional arthropod species lack a genus designation in the NCBI taxonomy. The sparseness of the mt16S database impacted the assignment of taxa actually detected in our surveys, as evidenced by the fact that the arthropod mt16S sequences reported by Mueller (2016) often had substantially better matches in our eDNA data than in the NCBI nt database.

**
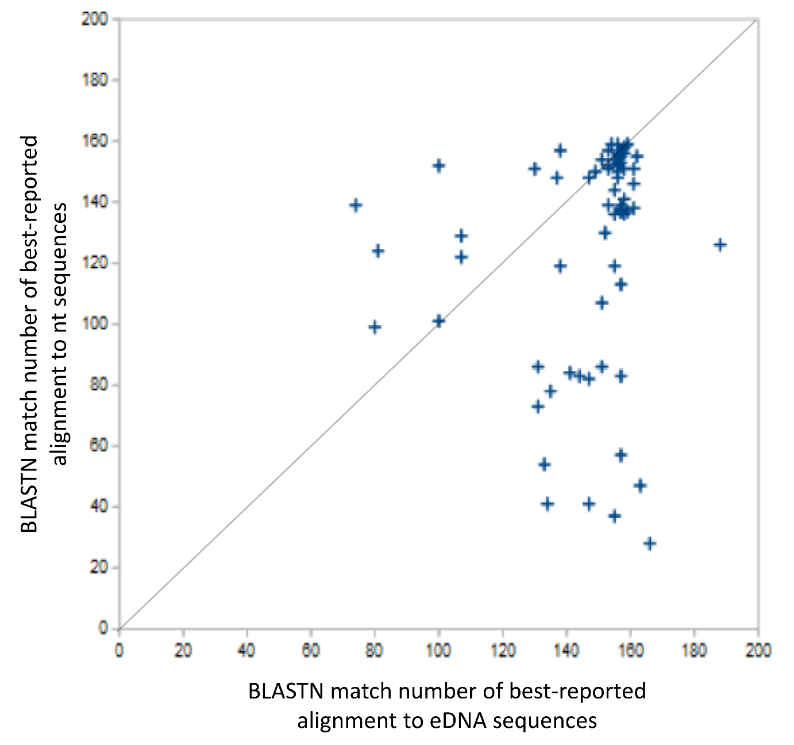
**

**Many mt16S voucher sequences had stronger alignments to eDNA data produced in this study than to sequences in the nucleotide database (nt) of the National Center for Biotechnology Information (NCBI).** Each point represents a pair of alignment scores (number of matched query bases) for an individual voucher sequence. Scores on the horizontal axis resulted from searching the voucher sequence against the representative sequences of all eDNA clusters, whereas scores on the vertical axis resulted from searching against nt. The diagonal line illustrates equal values by the two methods for reference.

Leave-one-out cross-validation (LOOCV) of the databases supported our use of a 0.9 scoring threshold for assigning environmental sequences. The genus-level error rate in the assignment of held-out queries at this threshold was 0.81% and 6.06%, respectively, for COI and mt16S.

**
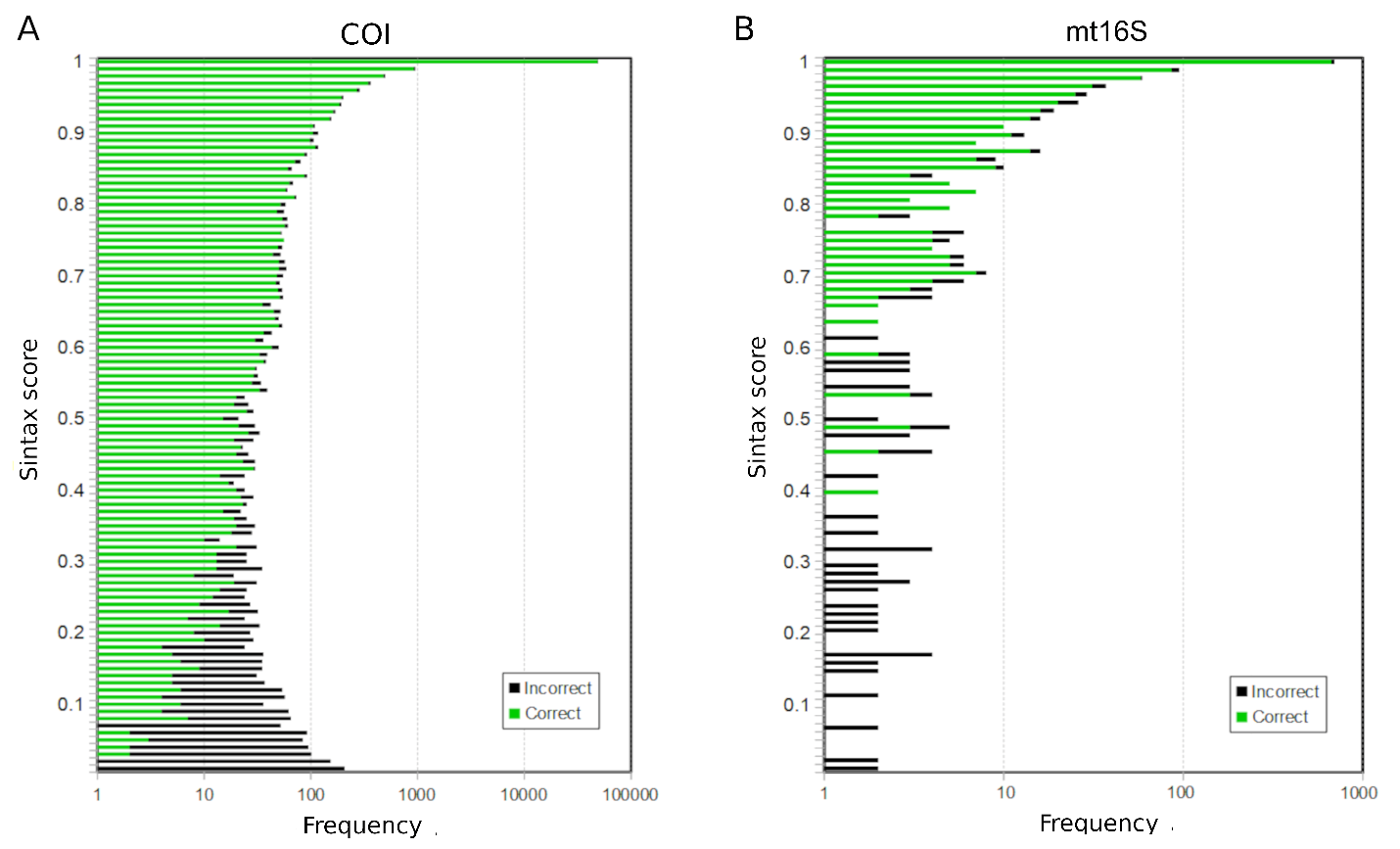
**

**Frequency of correct assignment of sequence-cluster representatives as a function of Sintax score in ‘leave-one-out’ cross-validation.** The number of correct and incorrect assignments are stacked in the barplot. A) Cross-validation of COI B) Cross-validation of mt16S.

To investigate the behavior of the mt16S assignment method as the number of reference sequences declined, we tabulated the SINTAX scores (which range from 0 to 1) assigned to correct and to incorrect LOOCV assignments, and plotted the average values by genus and by the number of reference sequences for each genus. We observed that a few mt16S genera were given higher assignment scores when incorrect than when correct, some of which are the same genera that we identified as conflicting in our voucher analysis (see below). We also observe a trend toward nonassignment as the number of reference sequences decline, rather than incorrect assignment, a preferred behavior.

**
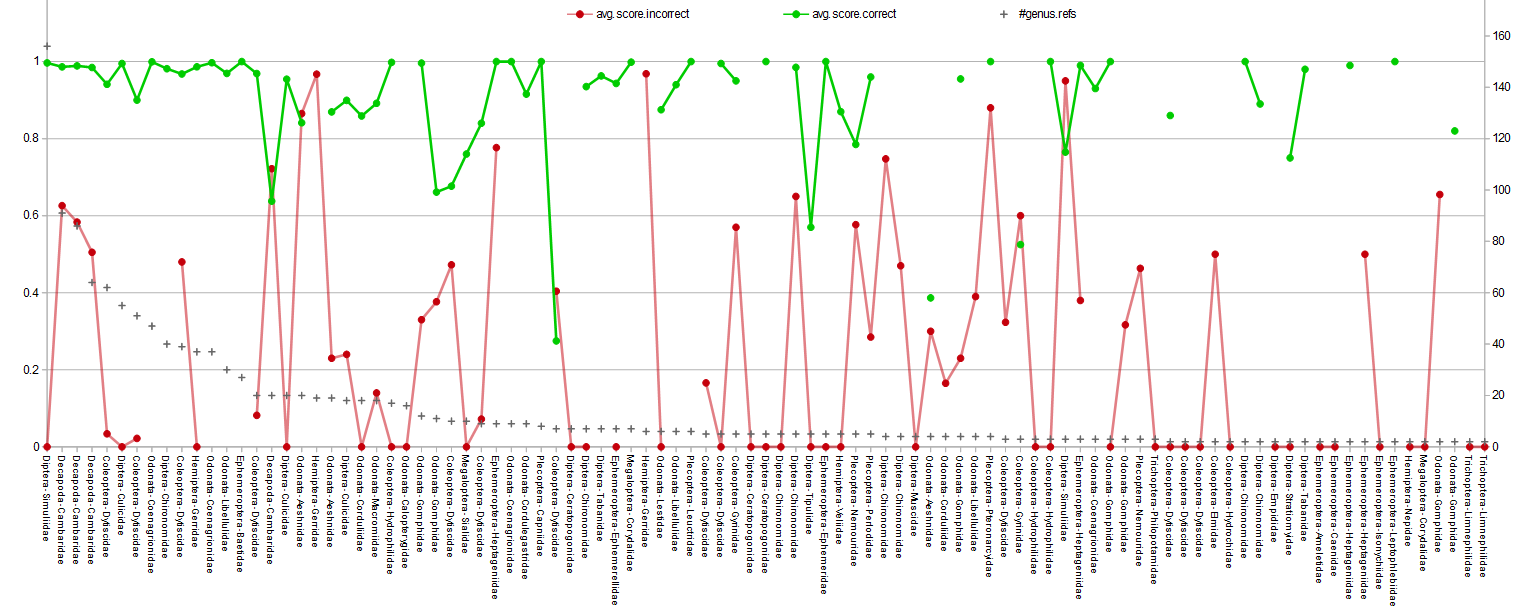
**

**Taxonomic assignment scores for held-out queries in leave-one-out validation, for genera in the mt16S database represented by more than one reference sequence.** The order and family of each genus is given on the horizontal axis.

SINTAX assignments of the mt16S voucher sequences reported by Mueller (2016) are summarized in **Supplemental File S7**. We limited our analysis to sequences from specimens manually classified to the genus level within phylum Arthropoda, and further to those with a top match in the nt database of at least 80% identity and a match score of 100. This requirement was imposed to ensure that the amplicon was legitimate and the manual assignment was reasonable. Voucher sequences assigned with SINTAX at a threshold of 0.9 matched manual assignments 17 of 22 times at the genus level (77.3%), whereas manual and SINTAX assignments matched at the family level in all cases. Several factors shaped assignment concordance that influenced the error rate in voucher assignments. One necessary factor was the absence of appropriate reference sequences for the manually assigned genus. Of the five test samples lacking genus-level representation in our curated database, four were unassigned by SINTAX, whereas the fifth (65_Erythemis) was assigned to a related genus (*Plathemis*).

The second factor we observed driving voucher misassignments was phylogenetic discordance between reference sequences and their taxonomic labels. Using the misassigned sequences 35_Calopteryx, 113_Faxonius, and 141_Stenonema as search queries, we generated neighbor-joining dendrogram of related sequences identified by BLASTN search (**Supplemental File S8**). All three trees show paraphyletic relationships, which for *Stenonema* and *Maccaffertium* is consistent with the findings of Zembrzuski and Anderson (2018) and for Calopteryx is consistent with Misof et al. (2016). Similarly, the water beetle genera *Stenelmis* and *Dubiraphia* are conflated for multiple vouchers, because only three reference sequences were available in total for these genera, two of which are identical within the barcode region yet assigned to different genera (accessions HQ629798.1 and DQ267445.1). Note that we are not arguing any phylogenetic hypothesis for these taxa with our data, only that available mt16S sequences as annotated cannot be expected to differentiate these genera for reasons extrinsic to the SINTAX method itself. We conclude from these validation steps that our curated database and the SINTAX method performed well in assigning the voucher sequences we obtained at a 0.9 threshold, except when there were no reference sequences or those sequences were phylogenetic discordant.

Literature cited:

Langmead B, Salzberg SL. 2012. Fast gapped-read alignment with Bowtie 2. Nature methods 9:357–359. https://doi.org/10.1038/nmeth.1923

Misof B, Anderson CL, Hadrys H. 2000. A phylogeny of the damselfly genus Calopteryx (Odonata) using mitochondrial 16S rDNA markers. Molecular phylogenetics and evolution 15:5–14. https://doi.org/10.1006/mpev.1999.0724

Maryland Department of Natural Resources. 2021. Maryland Biological Stream Survey Database. Available at www.dnr.maryland.gov/streams/Pages/dataRequest.aspx

Zembrzuski DC, Anderson FE. 2018. Clarifying the phylogenetic relationships and taxonomy of Stenonema, Stenacron and Maccaffertium, three common eastern North American mayfly genera. Molecular phylogenetics and evolution 128:212–220. https://doi.org/10.1016/j.ympev.2018.08.002
