# Supplementary material for: Assessing arthropod diversity metrics derived from stream environmental DNA: spatiotemporal variation and paired comparisons with manual sampling
Source: PeerJ. 2023 Mar 31;11:e15163. doi: 10.7717/peerj.15163 (PMC10069422; doi:10.7717/peerj.15163)

Supplemental File S8. Neighbor-joining dendrograms of three mt16S voucher sequences and top-matching accessions in the NCBI nucleotide database identified by homology search. The three search queries were incorrectly assigned at the genus level and the resulting phylogenies demonstrate discordance of NCBI reference sequences with assigned taxonomy. Trees were generated using a gamma rate distribution as described in the text. Bootstrap values are shown based on 1000 resampled replicates, but these are shown for information only and do not imply that these gene trees are sufficient evidence of phylogeny. A) *Calopteryx* (marked with red circles) and related genera B) *Faxonius* (marked with blue circles) and related genera C) *Stenonema* (marked with green circles) and related genera. See Supplemental File S6 for additional information.

A. *Calopteryx* mt16S reference sequences (red) are paraphyletic with multiple taxa

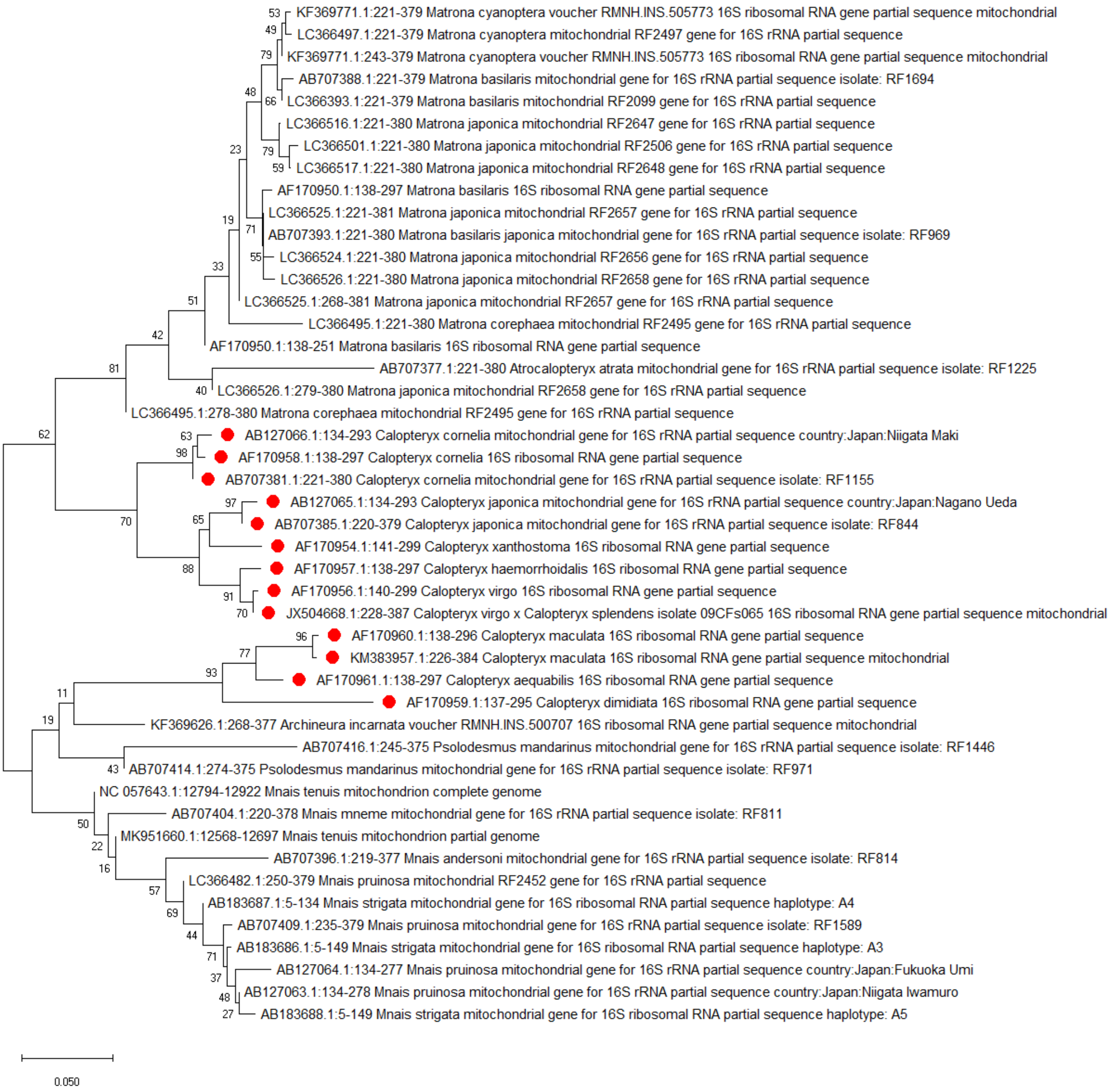

B. *Orconectes* mt16S reference sequences (blue) are paraphyletic with *Faxonius*

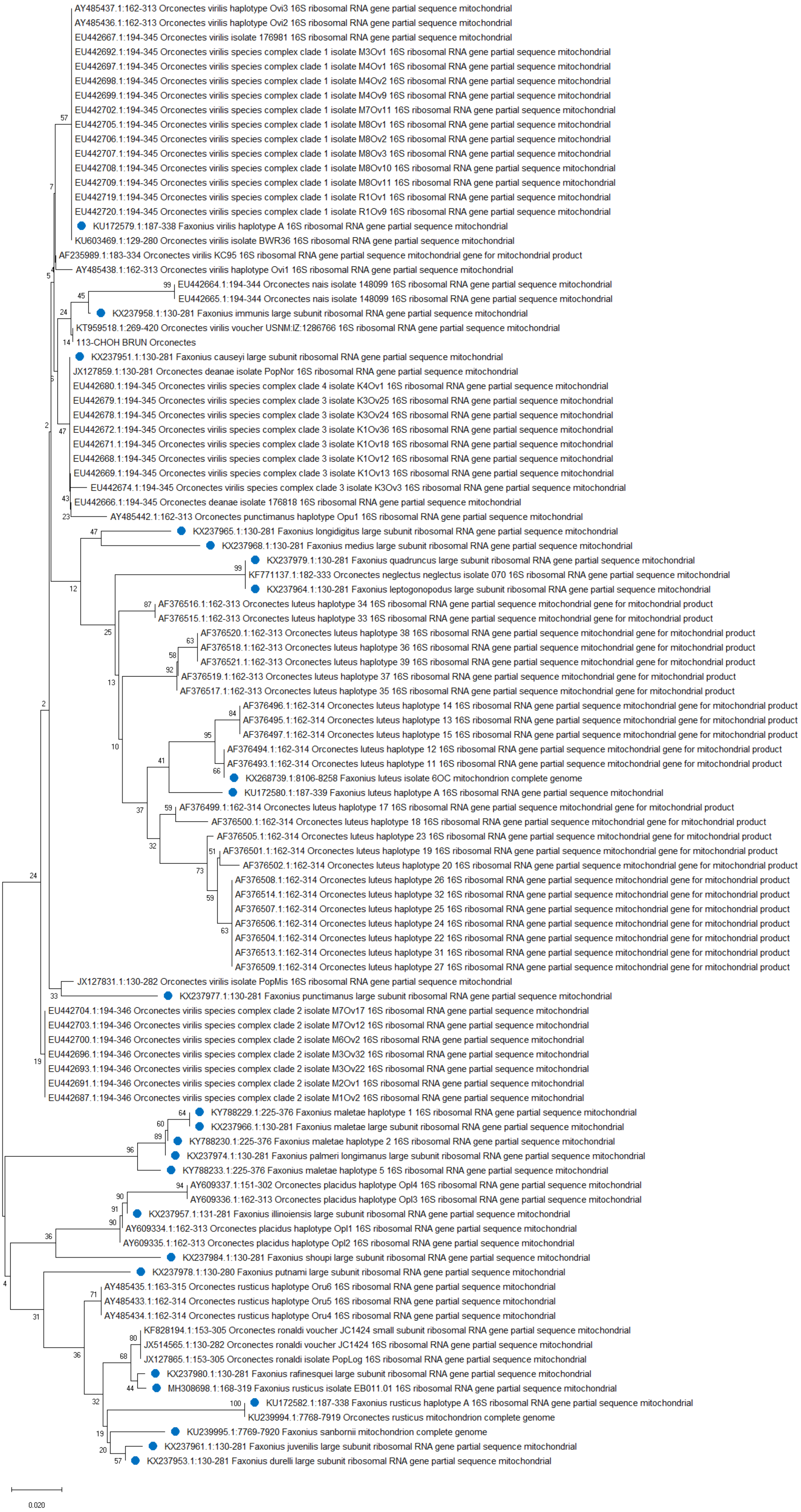

C. *Stenonema* mt16S reference sequences (green) are paraphyletic with *Maccaffertium*

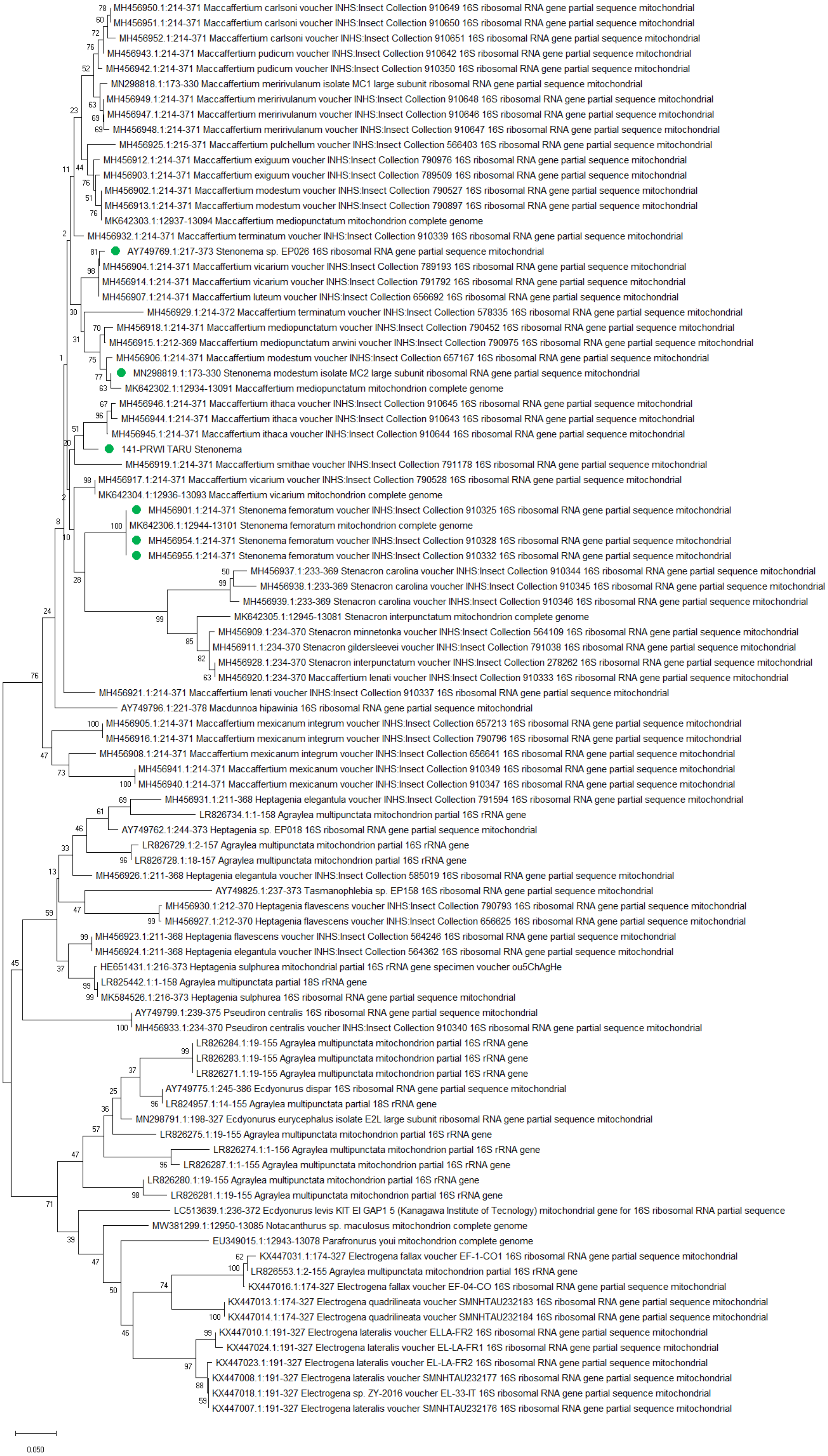

Supplement: Supplemental Information 8 — The three search queries were incorrectly assigned at the genus level and the resulting phylogenies demonstrate discordance of NCBI reference sequences with assigned taxonomy. Trees were generated using a gamma rate distribution as described in the text. Bootstrap values are shown based on 1,000 resampled replicates, but these are shown for information only and do not imply that these gene trees are sufficient evidence of organismal phylogeny. (A) Calopteryx (marked with red circles) and related genera (B) Faxonius (marked with blue circles) and related genera (C) Stenonema (marked with green circles) and related genera. mt16S, mitochondrial 16S ribosomal RNA gene. [file peerj-11-15163-s008.pdf]
