# Supplementary figures and images for: Assessing arthropod diversity metrics derived from stream environmental DNA: spatiotemporal variation and paired comparisons with manual sampling
Source: PeerJ. 2023 Mar 31;11:e15163. doi: 10.7717/peerj.15163 (PMC10069422; doi:10.7717/peerj.15163)

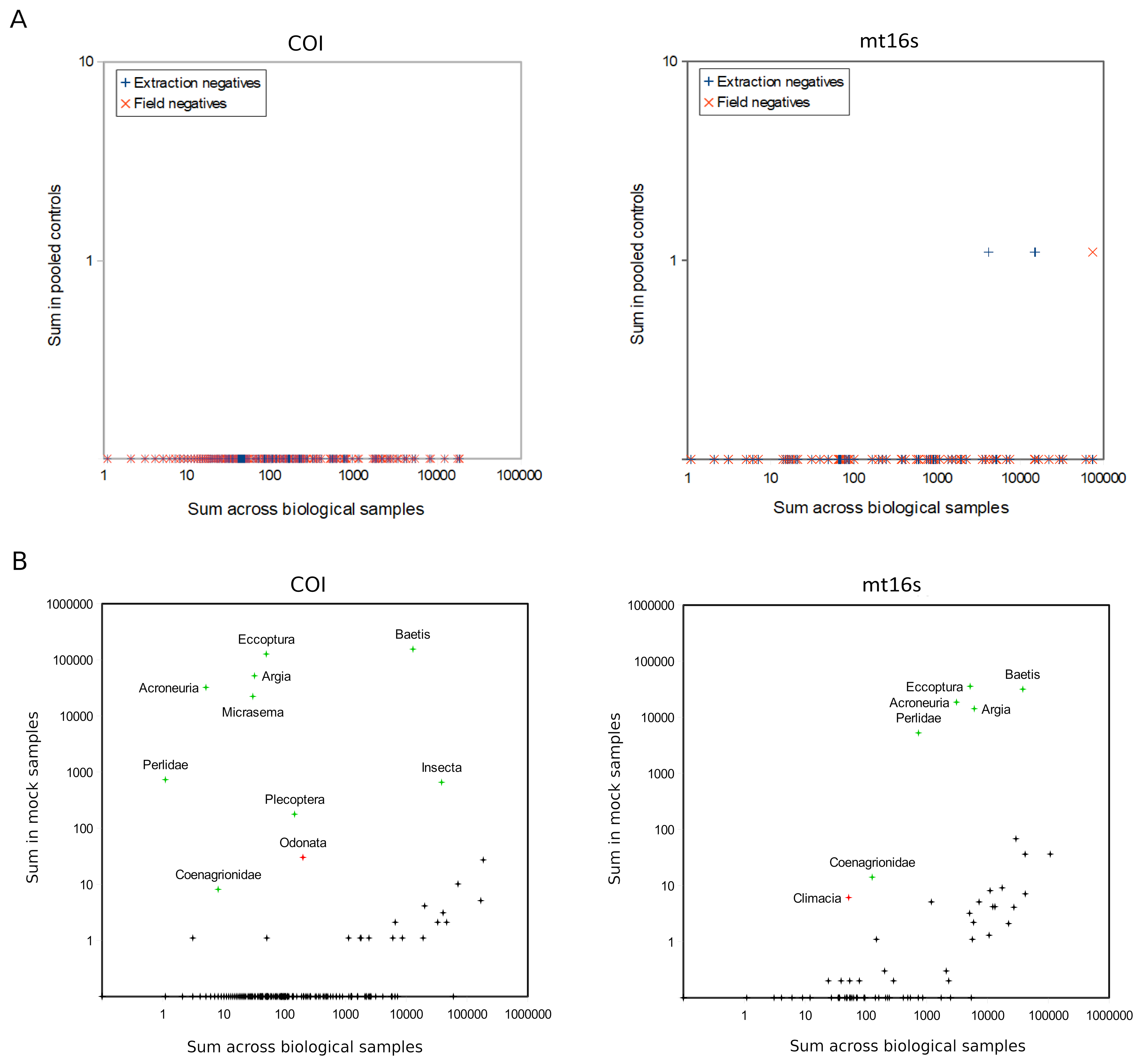

Supplement: Supplemental Information 13 — (A) Arthropod sequence in pooled field negative controls and laboratory extraction controls. Points indicate the number of reads of a given taxon summed across all biological samples vs. each negative control pool (all taxonomic ranks are shown). (B) Arthropod sequence in mock samples of known input composition. Green points indicate the number of reads for taxonomic assignments that are consistent with inputs, whereas black points are inferred to be crosstalk as they scale with total counts of a taxon at approximately 1 in 10,000. Red points are potential contaminants, in that they are visually elevated above the crosstalk distribution. COI, cytochrome c oxidase 1; mt16S, mitochondrial 16S. [file peerj-11-15163-s013.png]

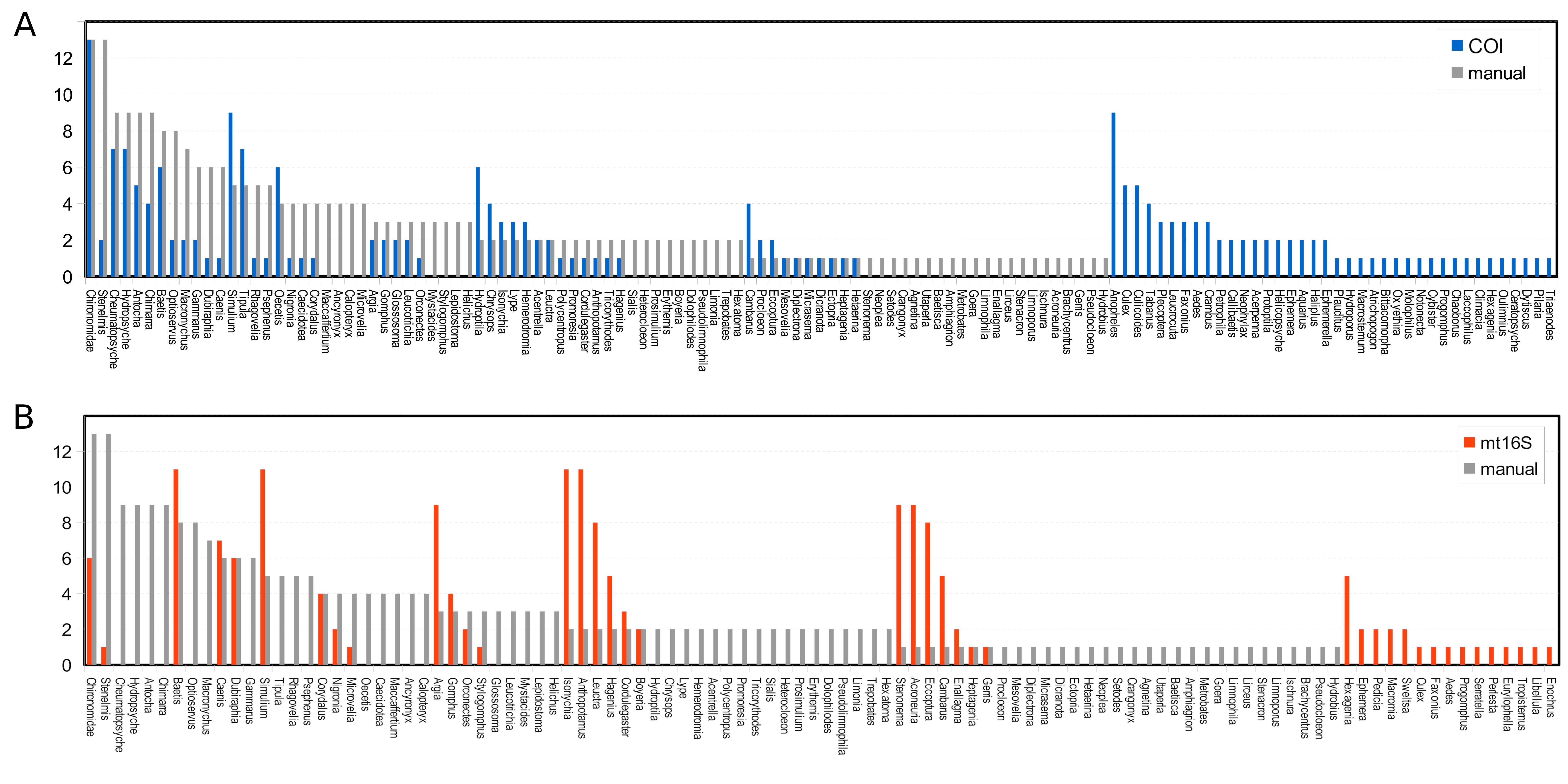

Supplement: Supplemental Information 14 [file peerj-11-15163-s014.png]

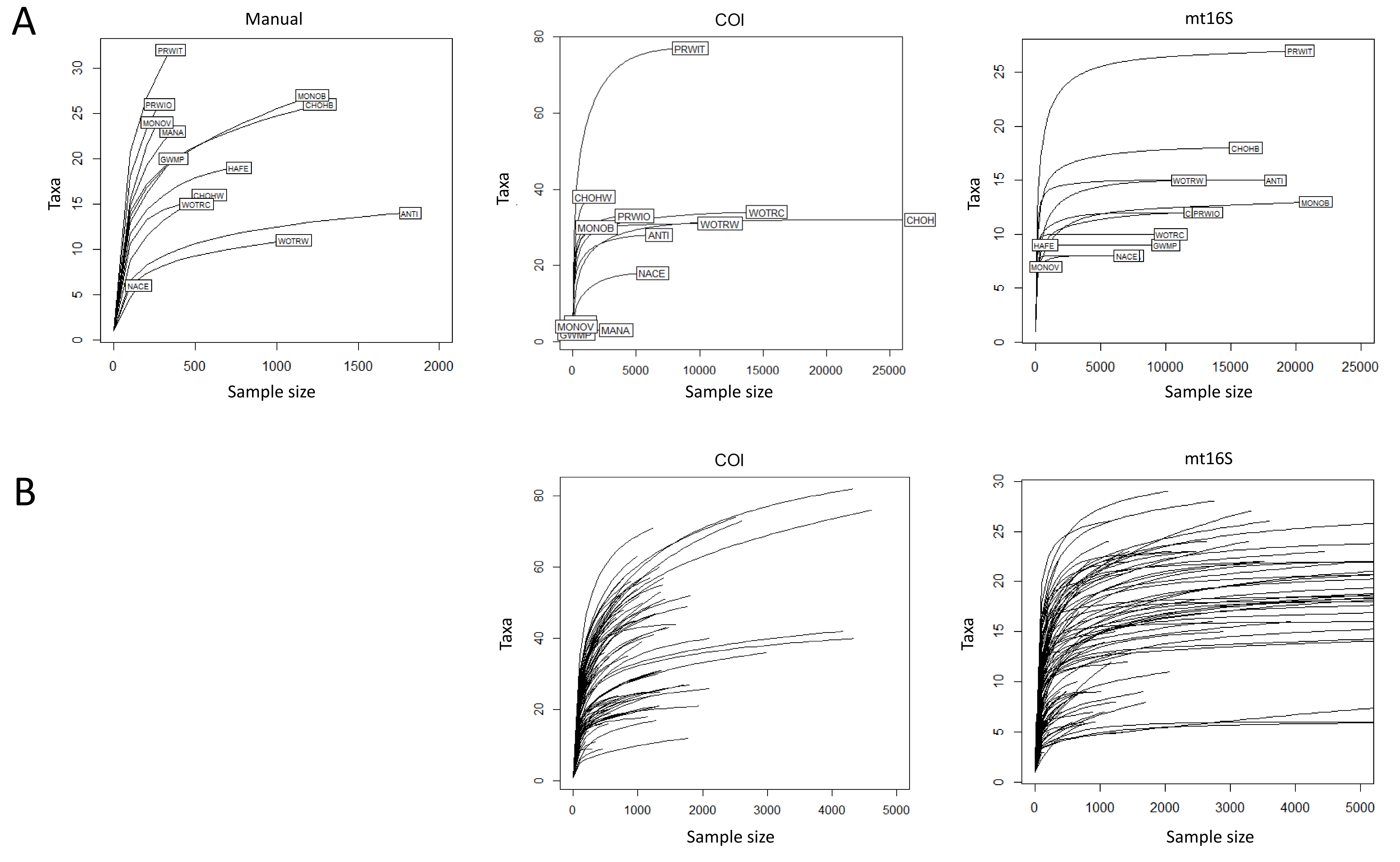

Supplement: Supplemental Information 15 — (A) Paired sampling with individual sites labeled. (B) Repeated sampling with individual samples unlabeled for clarity. The horizontal range is truncated at 5,000 sequence counts to facilitate a scale-matched comparison, since more reads are obtained at the mt16S locus on average. COI, cytochrome c oxidase 1; mt16S, mitochondrial 16S. [file peerj-11-15163-s015.png]

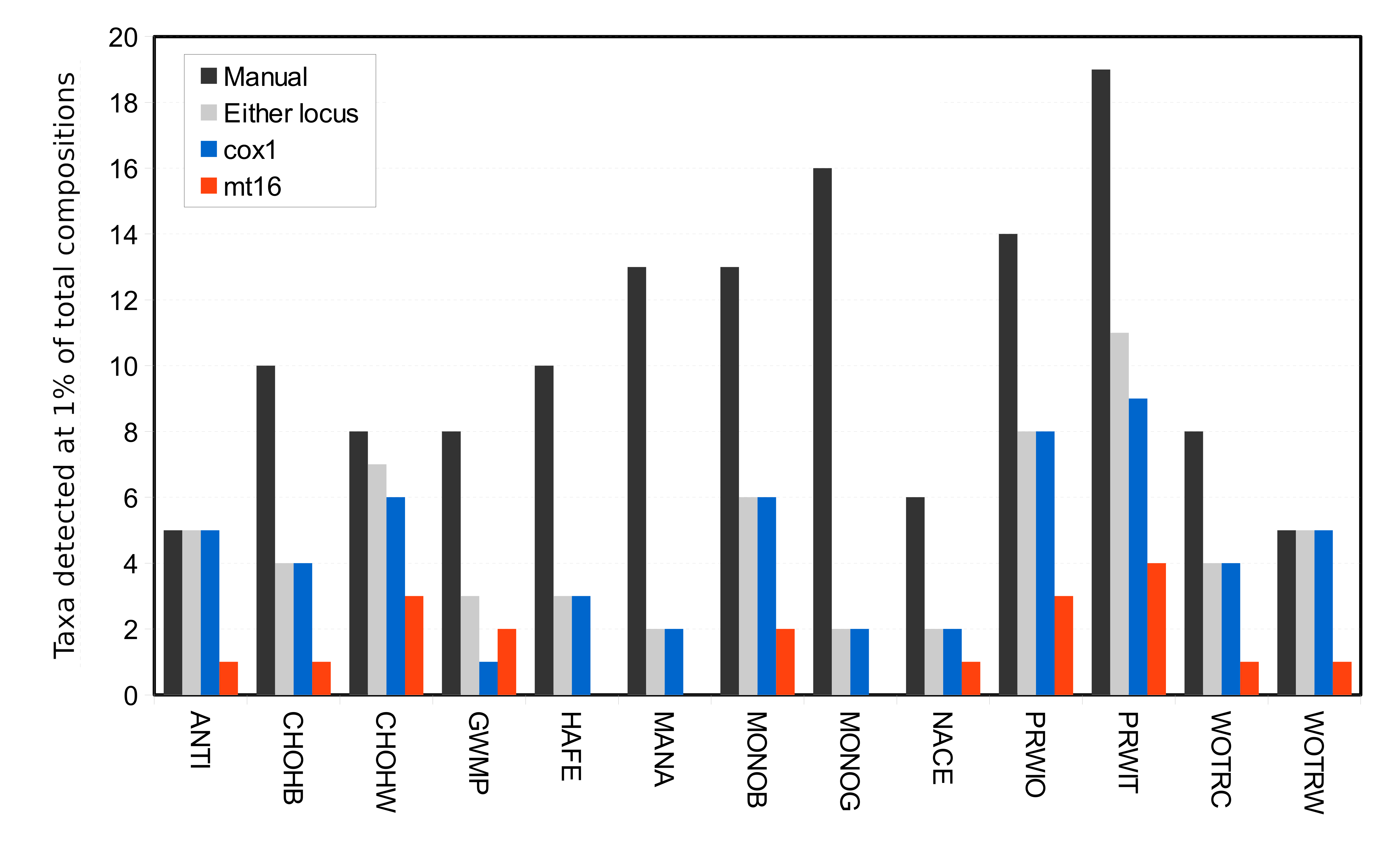

Supplement: Supplemental Information 16 [file peerj-11-15163-s016.png]

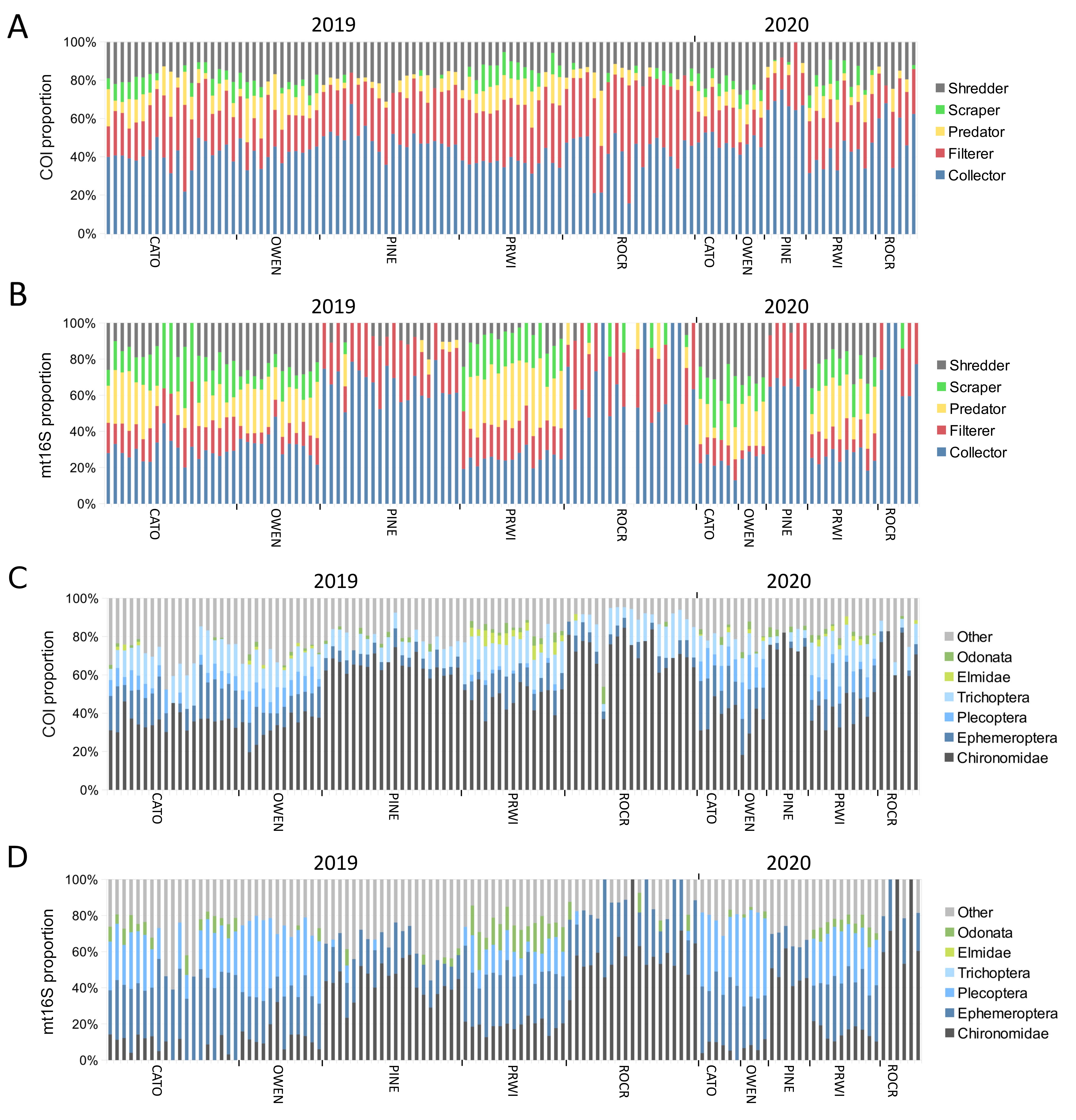

Supplement: Supplemental Information 17 — Reaches are listed in alphabetical order, but separately for summer 2019 and spring 2020 samples. The proportions of each group are relative, summing to 100% after excluding taxa assigned above the genus level (family level for Chironomidae) in the top panel or lacking a functional-group classification in the bottom panel. (A) Proportion of functional groups at COI. (B) Proportion of functional groups at mt16S. (C) Proportions of common arthropod indicator clades at COI. (D) Proportions of common arthropod indicator clades at mt16S. COI, cytochrome c oxidase 1; mt16S, mitochondrial 16S. [file peerj-11-15163-s017.png]

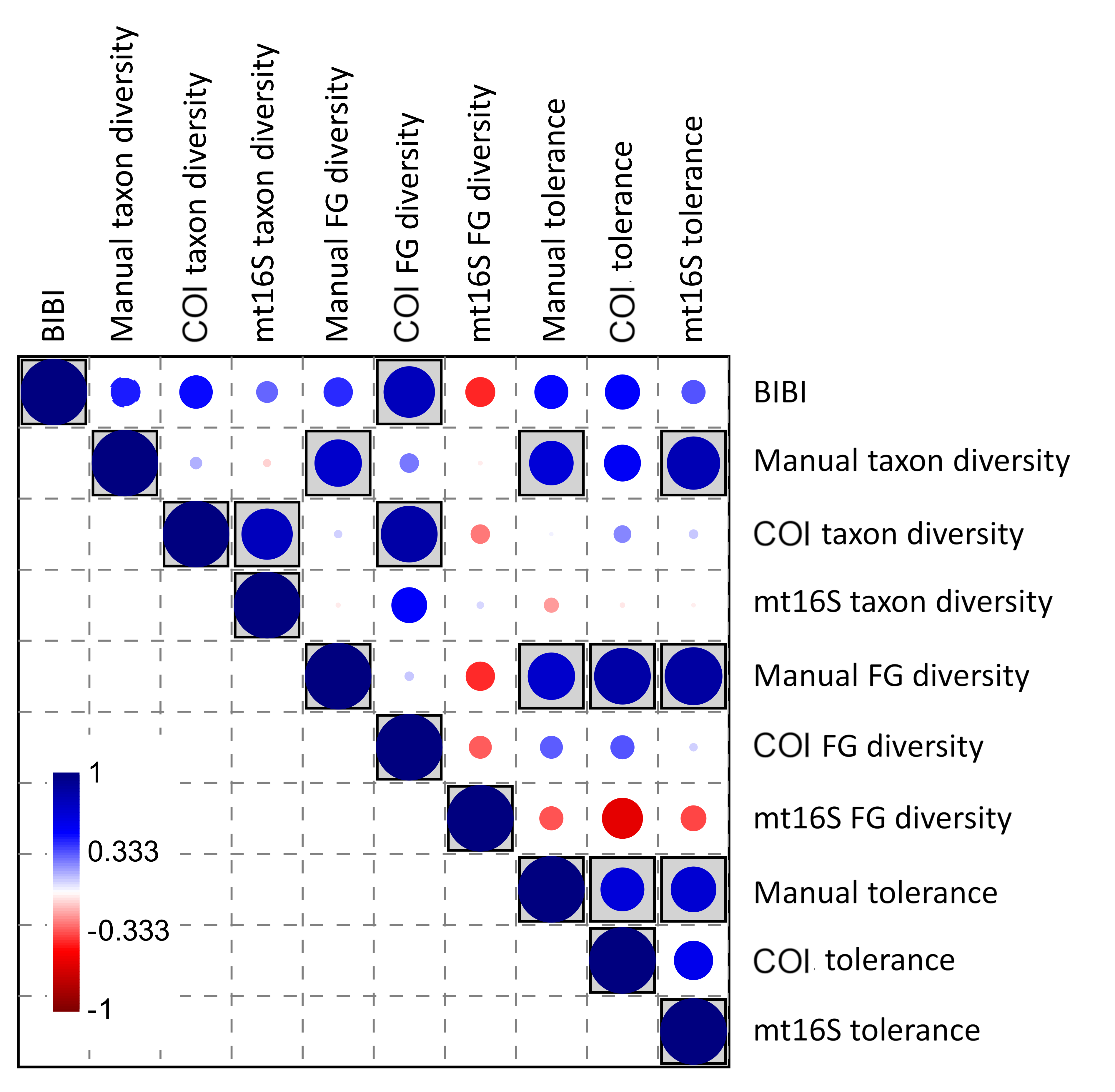

Supplement: Supplemental Information 18 — The size of the circle in each cell is proportional to the magnitude of the pairwise Spearman’s rank correlation coefficient, with blue circles indicating a positive correlation and red cells indicating a negative correlation coefficient. Gray-shaded cells indicate pairwise correlation coefficients that are significantly different than zero. FG, functional group. See File S12 for exact test results. COI, cytochrome c oxidase 1; mt16S, mitochondrial 16S. [file peerj-11-15163-s018.png]

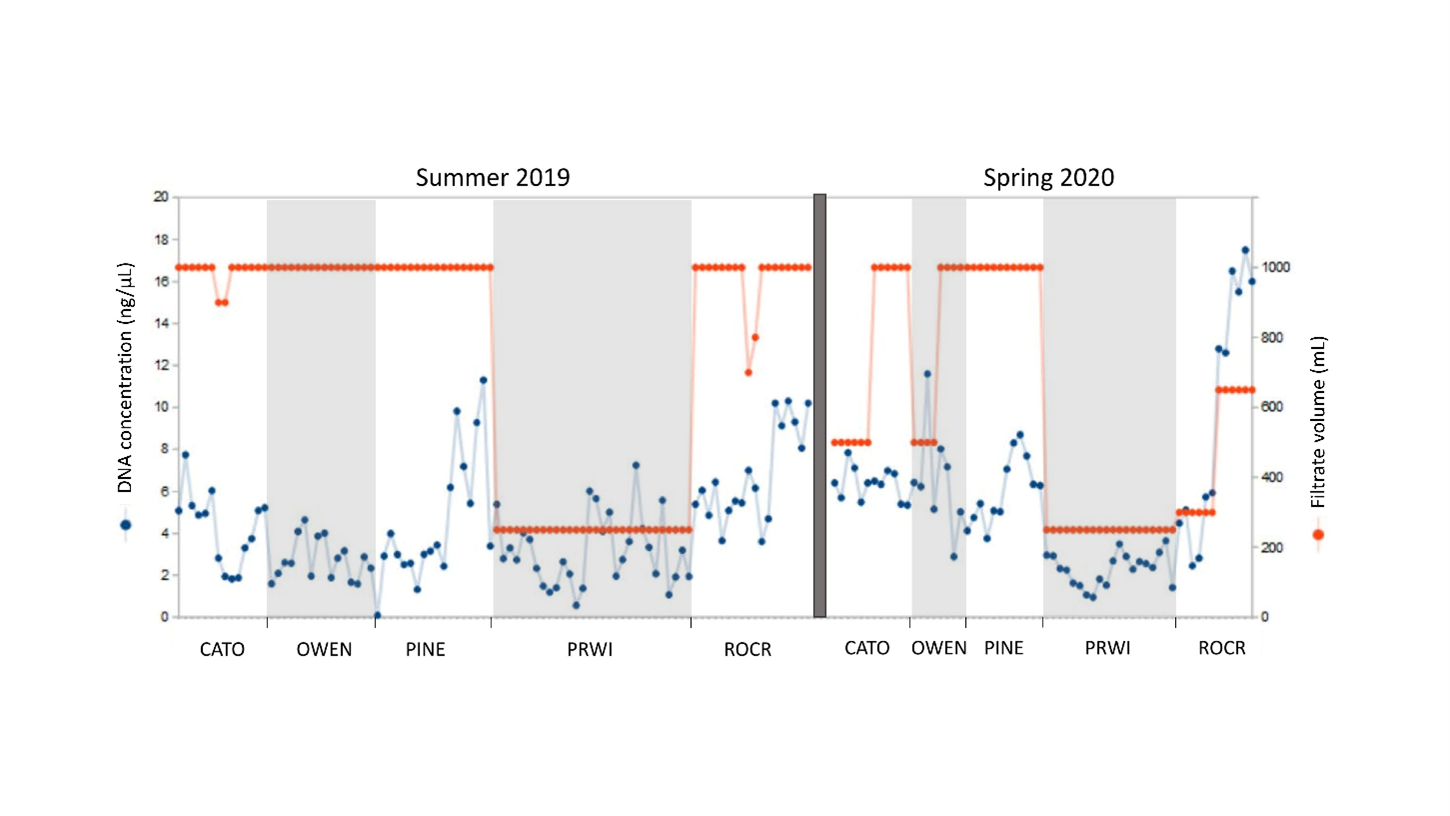

Supplement: Supplemental Information 19 [file peerj-11-15163-s019.png]

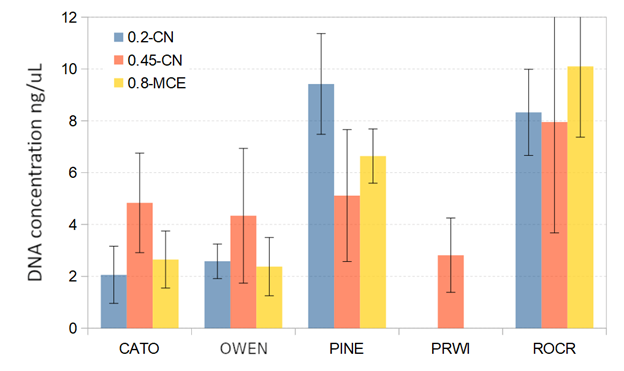

Supplement: Supplemental Information 20 — The three filter types tested were 0.2 micron cellulose nitrate (0.2 CN), 0.45 micron cellulose nitrate (0.45 CN), and 0.8 micron mixed cellulose esters (0.8 MCE). Lines indicate standard deviations for each reach-filter combination. The 0.2 CN and 0.8 MCE filters were not tested at PRWI. [file peerj-11-15163-s020.png]

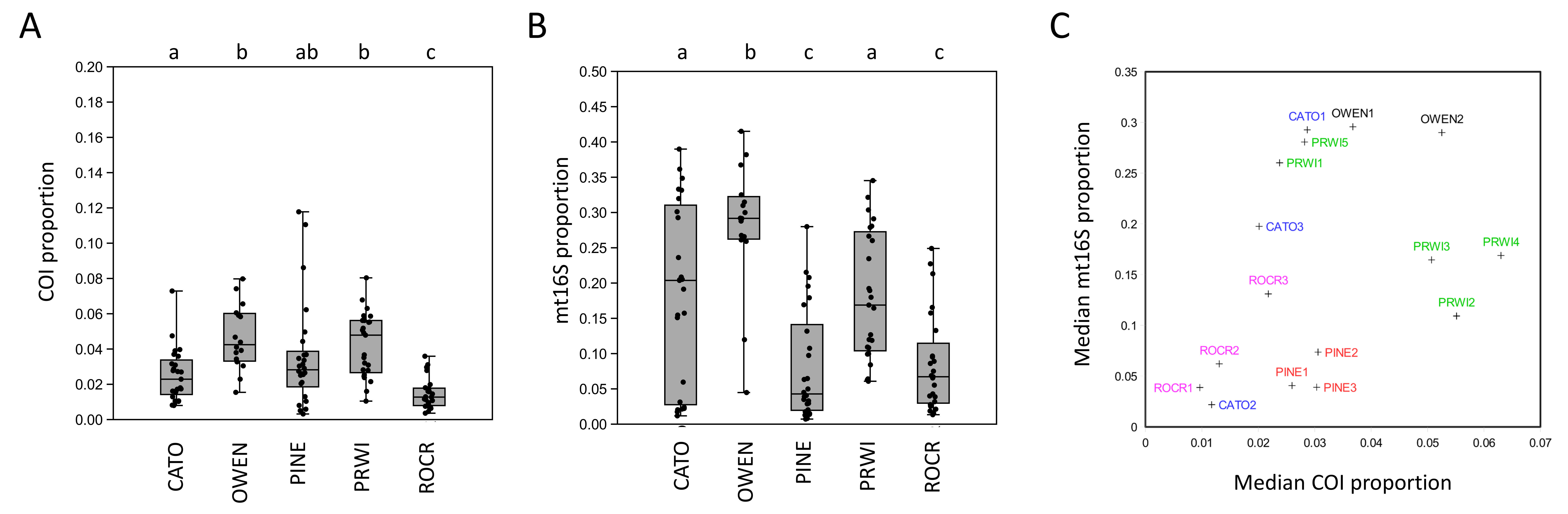

Supplement: Supplemental Information 21 — A significant difference in medians was assessed by Kruskal-Wallis test with letters indicating pairwise differences by Mann-Whitney U test after sequential Bonferroni adjustment of P-values. (A) Rates of arthropod read recovery at COI by reach. (B) Rates of arthropod read recovery at mt16S by reach. (C) Bivariate plot of median arthropod read rate by site within reaches. COI, cytochrome c oxidase 1; mt16S, mitochondrial 16S. [file peerj-11-15163-s021.png]

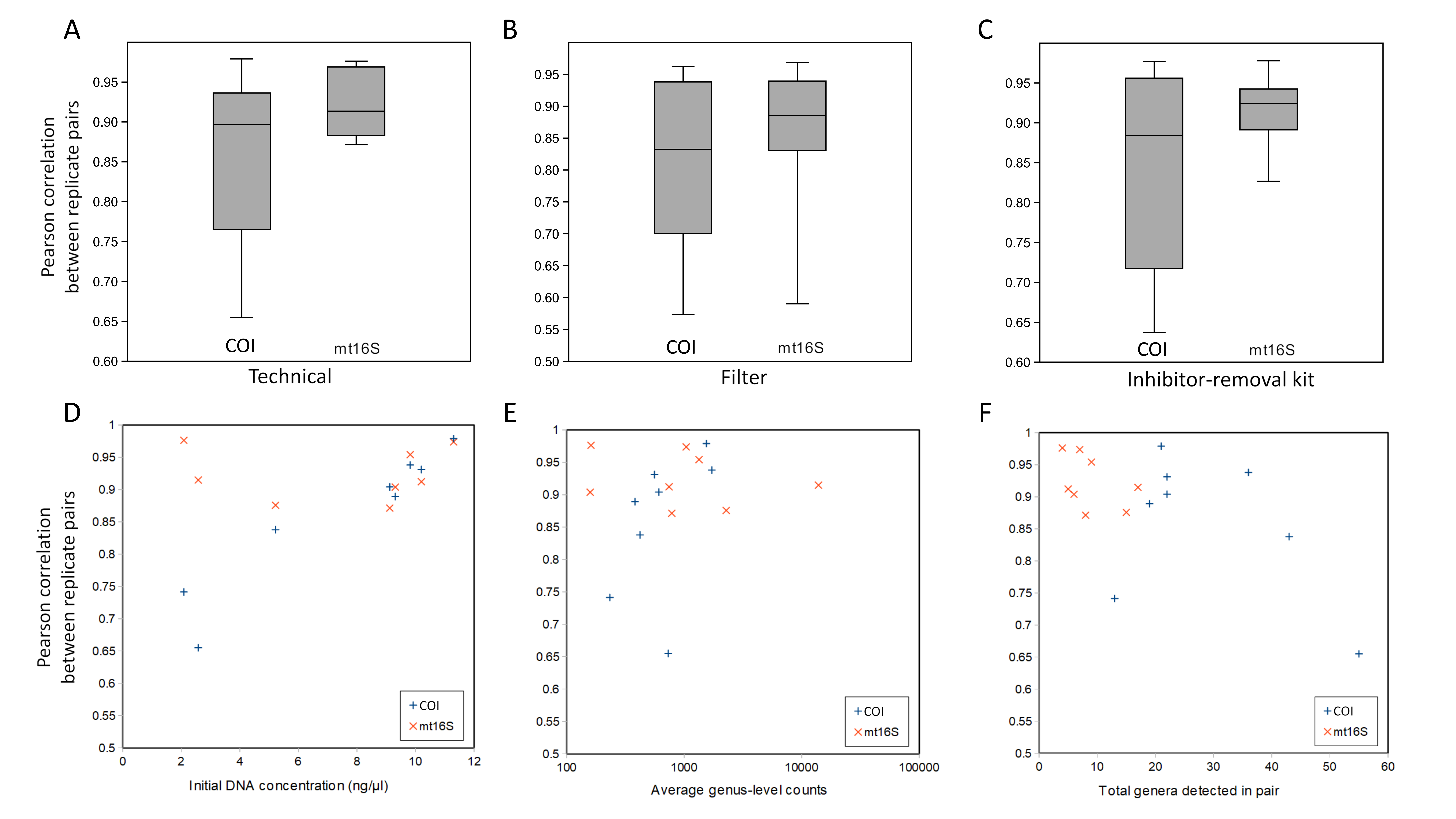

Supplement: Supplemental Information 22 — (A) Pairwise correlations of taxon compositions in strict technical replicates processed with the same protocol (n = 8). (B) Pairwise correlations of taxon compositions between technical replicates processed with different kits for removing PCR inhibition (n = 11, see text for details). (C) Pairwise correlation of taxon compositions in biological replicates obtained with one of three different filter types (n = 25, see text for details). All possible pairwise combinations were included. (D) Pearson correlation coefficients of taxon compositions between strict technical replicates as a function of extracted DNA concentration. (E) Pearson correlation coefficients of taxon compositions between strict technical replicates as a function of sequencing effort, measured as average number of reads assigned to arthropod genera. (F) Pearson correlation coefficients of taxon compositions between strict technical replicates as a function of the total richness of the two replicates. COI, cytochrome c oxidase 1; mt16S, mitochondrial 16S. [file peerj-11-15163-s022.png]

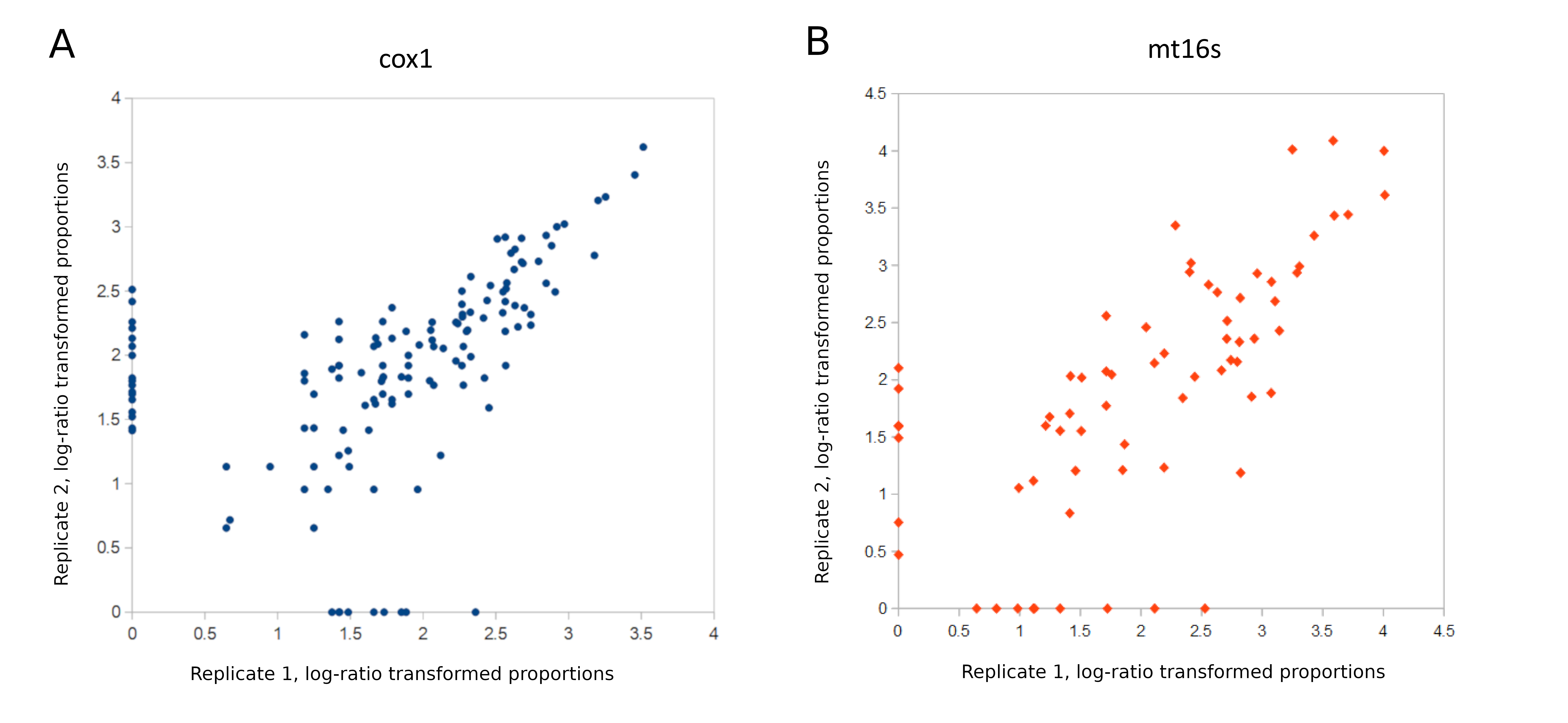

Supplement: Supplemental Information 23 — Each point represents the log-ratio transformed proportions of a taxon in two technical replicates, with all eight technical replicates plotted together. While taxon dropout occurs more frequently at lower values, log-transformed proportions are similar across the observed range of values when detected in both replicates. (A) COI transformed proportions (B) mt16S transformed proportions. COI, cytochrome c oxidase 1; mt16S, mitochondrial 16S. [file peerj-11-15163-s023.png]

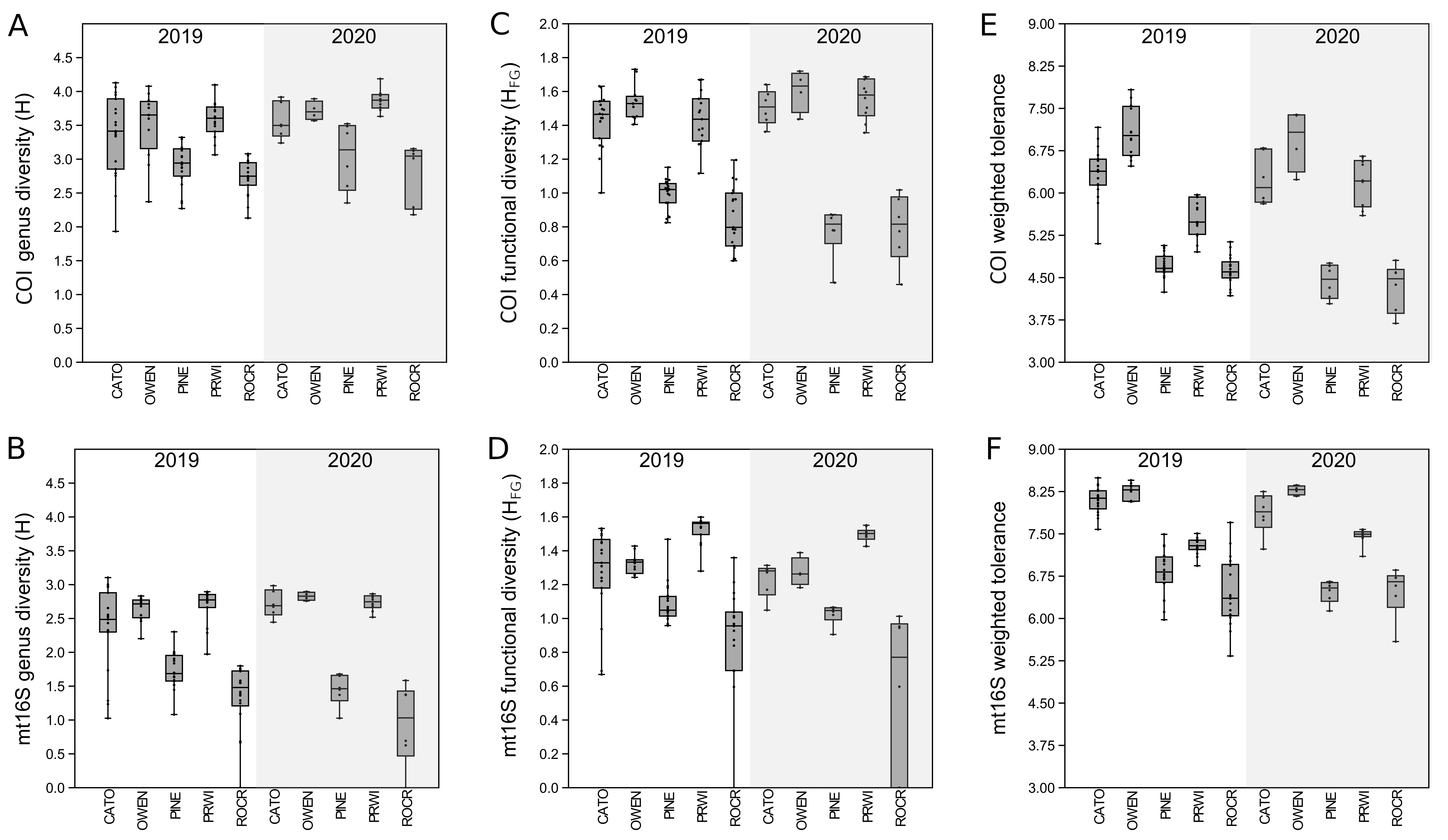

Supplement: Supplemental Information 24 — 2019 samples were collected in July, August, and September, whereas 2020 samples were collected from April to early June. Boxplots indicate range, mean, and quantiles for each site. Vertical-axis scale is fixed between loci for a given metric to facilitate comparison. (A) Shannon index of COI-detected arthropod genera (B) Shannon index of mt16S-detected arthropod genera (C) Shannon index of COI-detected functional groups (D) Shannon index of mt16S-detected functional groups (E) COI weighted tolerance score (F) mt16S weighted tolerance score. [file peerj-11-15163-s024.png]

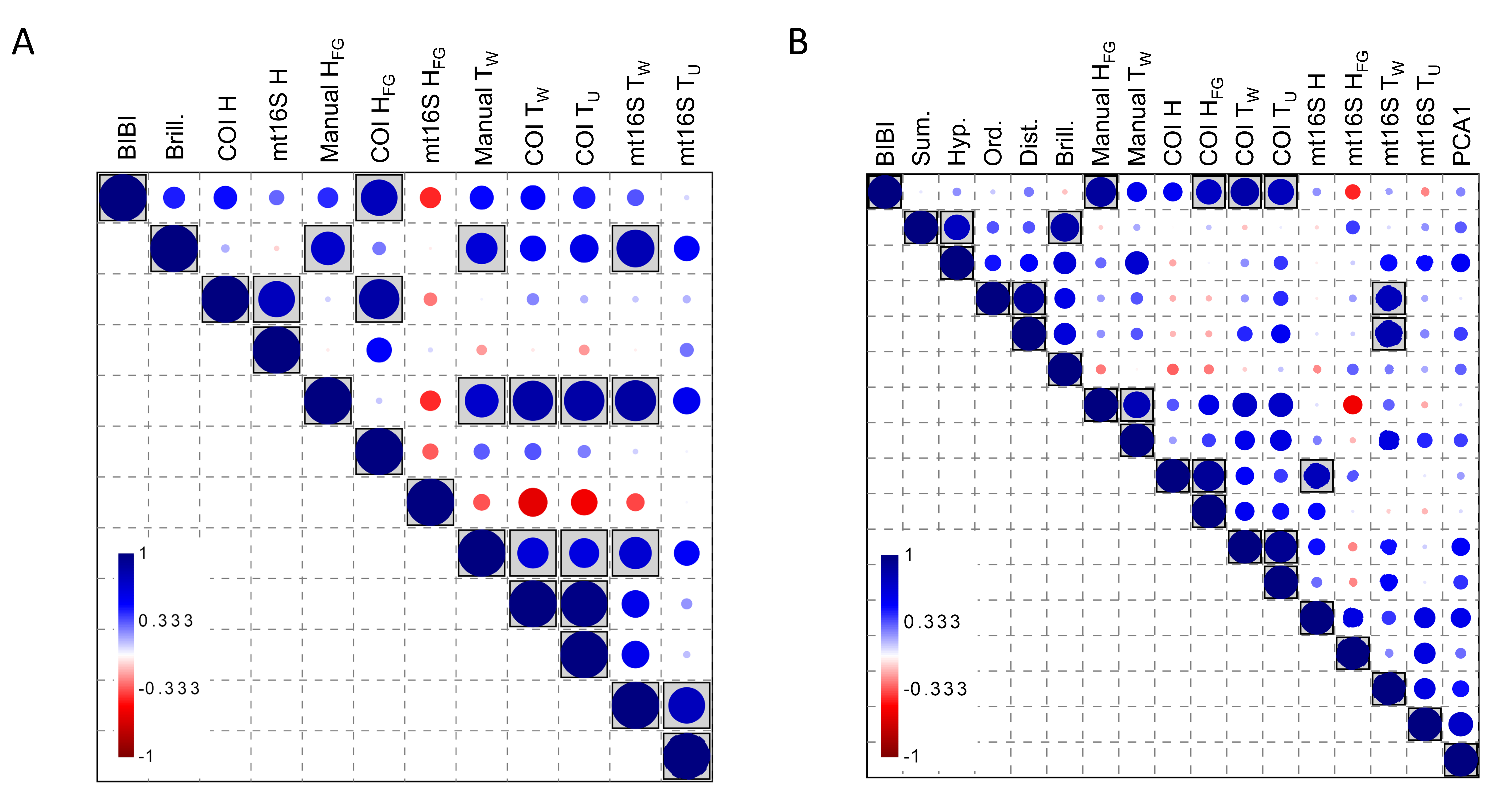

Supplement: Supplemental Information 25 — For unweighted tolerance, the scores for all genera present at greater than 1% of the sample sum (after log-ratio transformation) were averaged without weighting to obtain the sample-level tolerance score. (A) Spearman pairwise correlation matrix from Fig. S6 with additional columns for unweighted tolerance. (B) Spearman pairwise correlation matrix from Fig. 8 with additional columns for unweighted tolerance. BIBI, benthic invertebrate biological index; Brill, Brillouin’s index; H, Shannon index; HFG, Shannon index of functional groups; TW, weighted tolerance; TU, unweighted tolerance. COI, cytochrome c oxidase 1; mt16S, mitochondrial 16S. [file peerj-11-15163-s025.png]

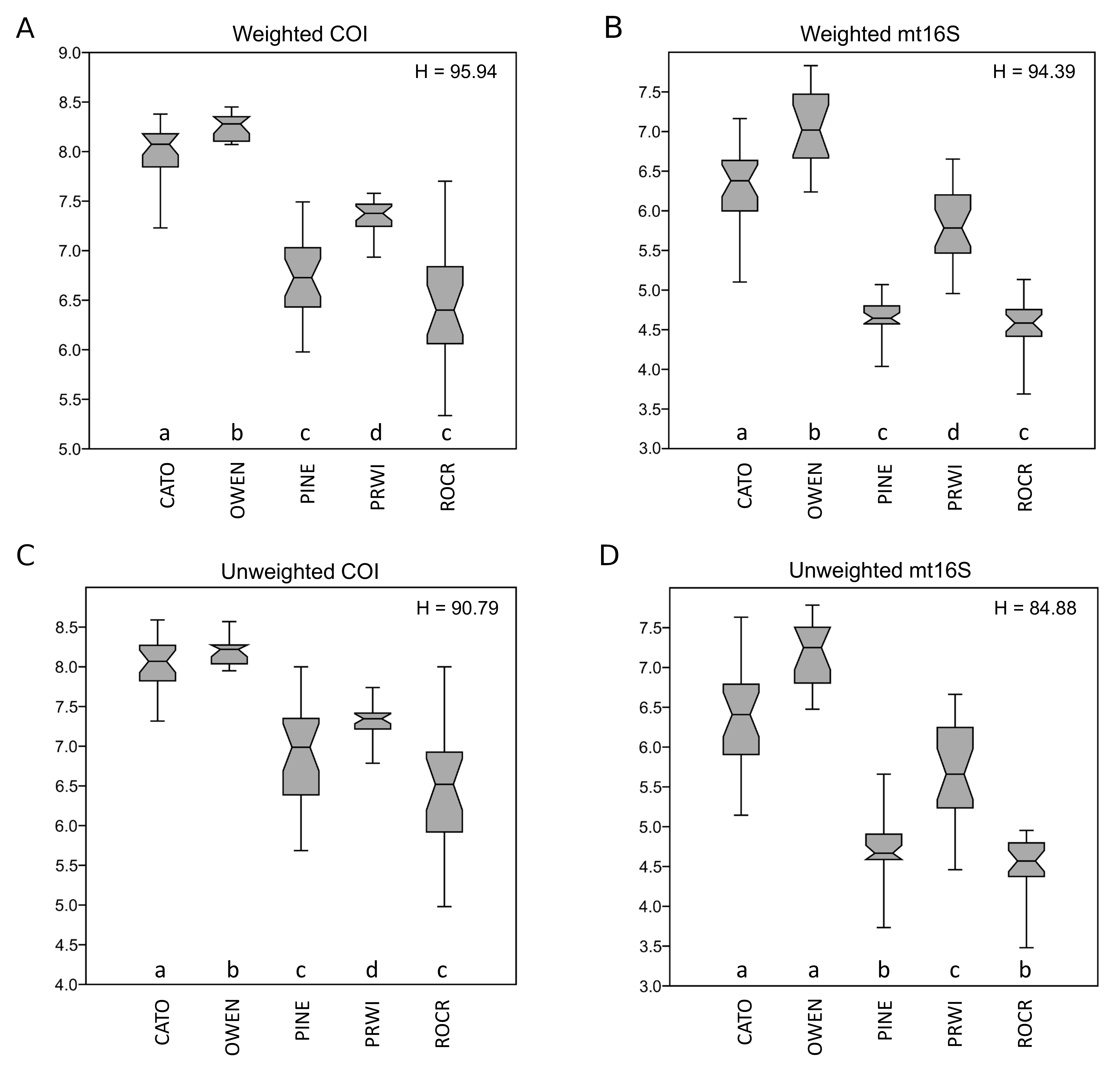

Supplement: Supplemental Information 26 — H is the test statistic of the Kruskal-Wallis test of median score among reaches, and reaches that share a lower-case letter are not significantly different by pairwise Mann-Whitney U test. See File S12 for test details. (A) Distribution of sample-level tolerance scores for COI-detected genera, weighted by transformed proportions. (B) Distribution of sample-level tolerance scores for mt16S-detected genera, weighted by transformed proportions. (C) Distribution of sample-level tolerance scores for COI-detected genera, equally weighted across those genera comprising >0.01 of the total after log-ratio transformation. (D) Distribution of sample-level tolerance scores for mt16S-detected genera, equally weighted across those genera comprising >0.01 of the total after log-ratio transformation. COI, cytochrome c oxidase 1; mt16S, mitochondrial 16S. [file peerj-11-15163-s026.png]

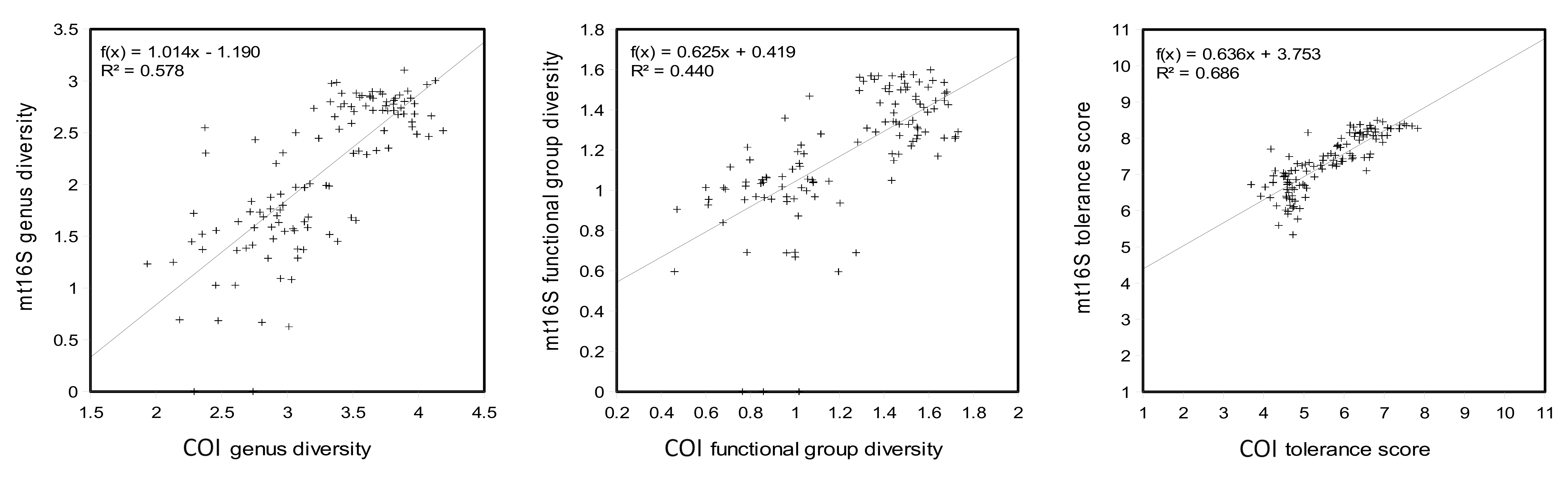

Supplement: Supplemental Information 27 — COI, cytochrome c oxidase 1; mt16S, mitochondrial 16S. [file peerj-11-15163-s027.png]
